# A Protein‐Centric Strategy Coupled with Match‐Between‐Run Glycoproteomics Enables Discovery of Robust Site‐Specific Glycan Biomarkers for Hepatocellular Carcinoma

**DOI:** 10.1002/advs.202516299

**Published:** 2026-02-12

**Authors:** Lei Liu, Taiheng Ma, Qi Liu, He Zhu, Zheng Fang, Jiahong Ma, Ting Yu, Yan Wang, Jiahua Zhou, Xiaoyan Liu, Yaqian Li, Zhimou Guo, Xinmiao Liang, Mingming Dong, Deguang Sun, Mingliang Ye

**Affiliations:** ^1^ State Key Laboratory of Medical Proteomics, CAS Key Laboratory of Separation Science For Analytical Chemistry Dalian Institute of Chemical Physics Chinese Academy of Sciences Dalian China; ^2^ University of Chinese Academy of Sciences Beijing China; ^3^ Division of Hepatobiliary and Pancreatic Surgery, Department of General Surgery The Second Hospital of Dalian Medical University Dalian China; ^4^ MOE Key Laboratory of Bio‐Intelligent Manufacturing School of Bioengineering Dalian University of Technology Dalian China

**Keywords:** biomarker, hepatocellular carcinoma, machine learning model, N‐glycoproteomics, protein‐centric, site‐specific glycans

## Abstract

Dysregulated protein glycosylation is a hallmark of cancer, and systematic investigation of glycosylation patterns is crucial for identifying biomarkers. However, current glycoproteomic studies are constrained by the limited quantification power of single MS files and focus on dysregulated glycopeptides while neglecting the underlying glycoproteins. To address this, we proposed a protein‐centric strategy to prioritize proteins susceptible to aberrant glycosylation, aiming to uncover previously overlooked cancer‐associated proteins. In this study, we analyzed 200 samples via quantitative glycoproteomics on an integrated platform. Notably, the Glyco‐Decipher software's new match‐between‐run scheme was applied in a large‐scale serum‐based HCC cohort study to enhance single‐shot intact glycopeptide profiling, boosting detection of significantly dysregulated site‐specific glycans 4.8‐fold compared to conventional method. The protein‐centric strategy identified 26 glycoproteins, with Fibronectin emerging as a top diagnostic performer. Specifically, the N1007_H5N4S2 on Fibronectin exhibited excellent diagnostic performance for HCC, achieving an AUC value of 0.917. Furthermore, a machine learning model integrating N1007_H5N4S2 on Fibronectin and N107_H9N3 on Alpha‐1‐antitrypsin yielded AUC values of 0.950/0.973 (HCC), 0.976/0.922 (TNM‐I HCC), and 0.948/0.867 (AFP‐negative HCC) in two cohorts, respectively. These findings demonstrated the effectiveness of the protein‐centric strategy in identifying robust biomarkers, highlighting the potential of site‐specific glycans for improving HCC diagnosis.

## Graphical abstract

In this study, a highly robust N‐glycoproteomics (HRN) platform with a newly developed match‐between‐run scheme is used for biomarker discovery. This study proposes a protein‐centric strategy to prioritize proteins susceptible to aberrant glycosylation. These dysregulated site‐specific glycans demonstrate distinct glycosylation pattern in the up‐/down‐regulated groups. The Fibronectin protein emerges as a top and robust diagnostic performer of hepatocellular carcinoma.

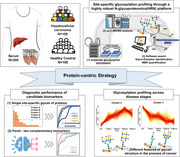

## 1 Introduction

Hepatocellular carcinoma (HCC) is a leading cause of cancer‐related mortality worldwide, primarily due to late‐stage diagnosis and limited treatment options. The routinely applied imaging and histopathological evaluation strategies often fail to detect early‐stage HCC, where timely intervention could markedly improve outcomes. Accordingly, identifying non‐invasive and patient‐friendly biomarker candidates is imperative to advance early HCC detection. Protein glycosylation, a pivotal post‐translational modification, regulates critical biological processes including cell‐cell interactions [1], immune modulation [2, 3], and signal transduction [4], with its dysregulation recognized as a hallmark of cancer. Aberrant glycosylation, including increased branching, fucosylation and sialylation, is prominent in cancer and well‐associated with tumor growth, metastasis and immune evasion [1]. Currently, most tumor biomarkers approved for clinic are serum glycoproteins, including cancer antigen 125 (CA125, MUC16) for ovarian cancer [5], prostate‐specific antigen (PSA) for prostate cancer [6], alpha‐fetoprotein (AFP) and its core‐fucosylated isoform AFP‐L3 for HCC [7, 8]. Moreover, the improved specificity of core‐fucosylated AFP over total AFP levels in HCC detection highlights the significance of site‐specific glycans in biomarker discovery. However, as a significant subset of HCC patients are AFP‐negative, discovering novel biomarker candidates with aberrant glycosylation is critical to enhance HCC detection.

Two primary strategies have been employed to screen site‐specific glycan biomarkers for HCC. The first strategy employs glycoproteomics approaches for analyzing glycopeptides derived from almost all serum proteins, and these studies have established foundational methods for HCC biomarker discovery [9, 10, 11, 12, 13]. The second strategy, which targets specific glycoproteins (e.g., Haptoglobin [14, 15, 16, 17, 18, 19] and IgG [20, 21]) purified from serum, enables the detection of low‐abundance glycopeptides derived from the purified glycoproteins but fails to identify novel glycoproteins. While quantitative glycoproteomics using data‐dependent acquisition (DDA) has advanced glycosylation research in diseases, [9, 22, 23, 24] significant challenges remain: the stochastic nature of DDA, the wide dynamic range of protein glycosylation, and the limited glycopeptide spectrum interpretation rates collectively result in substantial missing values that hinder sensitive biomarker detection. For instance, Liu et al. quantified 19,697 unique site‐specific glycans across 140 clinical samples, yet only 179 (0.91%) maintained non‐missing values. Regarding the missing value issue, quantified glycopeptides with 70% valid values in at least one group in the discovery cohort were reserved for downstream statistical analysis, and then the missing values of the retained intact N‐glycopeptides were treated with zero‐imputation. Consequently, only 933 quantified site‐specific glycans (4.74%) were used for subsequent t‐test differential analysis [22]. This led to a significant loss of data. Furthermore, most glycoproteomic studies focus narrowly on individual dysregulated glycopeptides quantified by shotgun proteomics while neglecting their parent proteins. In reality, glycosylation exerts widespread yet preferential effects on proteins during disease onset and progression. From the perspective of glycoproteomics, if multiple glycopeptides derived from a given protein exhibit aberrant glycosylation in cancer, the protein can be considered susceptible to aberrant glycosylation. Notably, proteins with higher numbers of dysregulated site‐specific glycans are relatively rare, whereas proteins with single dysregulated site‐specific glycans predominate. This disparity highlights the importance of prioritizing proteins susceptible to dysregulated glycosylation, which could reveal novel roles in disease pathogenesis and uncover distinctive glycosylation dysregulation patterns.

Match‐Between‐Run (MBR) has been incorporated as an effective imputation strategy in several glycopeptide analysis platforms [25, 26, 27], enabling the generation of more highly confident quantitative results and has been applied in subsequent studies [28]. Recently, GlyPep‐Quant software which enables MBR across files and library‐based virtual MBR was developed by our group [29] and integrated in Glyco‐Decipher [30]. In GlyPep‐Quant, a two‐step elution profile extraction method was developed to precisely and efficiently locate the matched profiles and a pre‐trained random forest machine learning model was employed for the confidence assessment of the extracted elution profiles. GlyPep‐Quant was demonstrated to enable the quantification of more glycopeptides without any missing values than other state‐of‐the‐art tools. In this study, we propose a novel protein‐centric strategy integrated with the MBR glycoproteomics workflow. This approach aims to: (1) enhance quantitative coverage by reducing missing values through MBR‐based imputation; (2) prioritize proteins with multiple dysregulated site‐specific glycans to uncover understudied candidates; and (3) develop robust biomarkers or biomarker panels for early and AFP‐negative HCC detection. In this study, the MBR scheme significantly reduced missing values from 85.2% to 19.4% and increased quantifiable site‐specific glycans 6.2‐fold in the dataset of the discovery cohort. This enabled the detection of 1,038 dysregulated site‐specific glycans, a 4.8‐fold improvement over conventional method. The protein‐centric strategy prioritized 26 glycoproteins with ≥10 dysregulated site‐specific glycans in the discovery cohort, and these dysregulated site‐specific glycans accounted for 55.6% of the total dysregulated site‐specific glycans. Notably, Fibronectin emerged as a top performer: the site‐specific glycan N1007_H5N4S2 exhibited excellent diagnostic performance (AUCs: 0.917/0.946 in the discovery/validation cohorts), outperforming AFP even for early‐stage (TNM‐I, AUCs: 0.896/0.919) and AFP‐negative (AUCs: 0.894/0.908) HCC diagnosis. A machine learning model combining Fibronectin_N1007_H5N4S2 and Alpha‐1‐antitrypsin_N107_H9N3 achieved even higher AUCs (0.950/0.973 for HCC, 0.976/0.922 for TNM‐I, 0.948/0.867 for AFP‐negative cases). This study demonstrates the power of MBR‐enhanced glycoproteomics and the protein‐centric screening strategy in discovering robust biomarkers, addressing critical unmet needs in early and AFP‐negative HCC diagnosis.

## 2 Results

### 2.1 Study Design and Clinical Characteristics of Serum Samples

**FIGURE 1:**
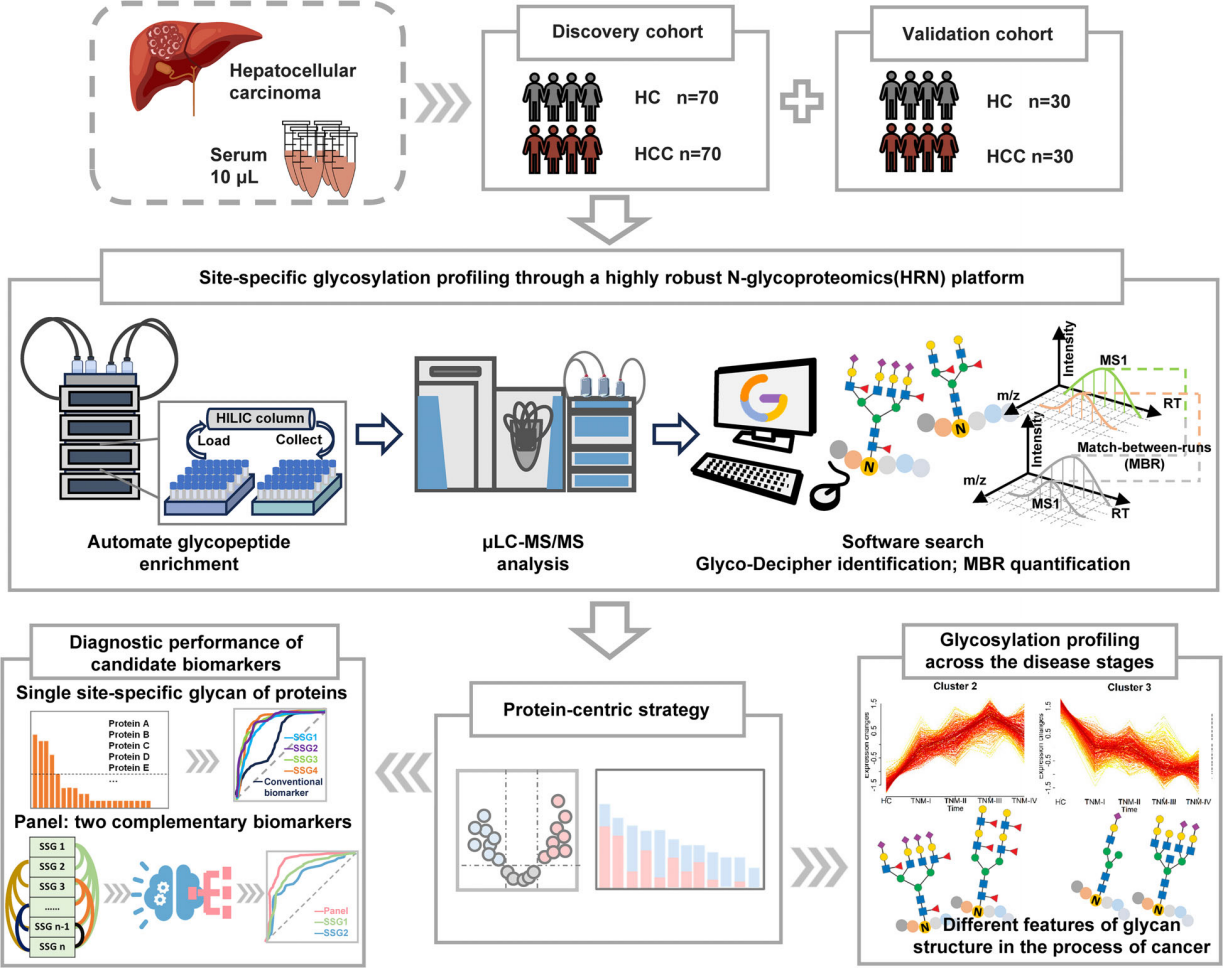
Workflow for discovering serum site‐specific glycan (SSG) biomarkers on proteins susceptible to dysregulated glycosylation using a protein‐centric strategy. In this platform, serum N‐glycopeptides were enriched through an automated HPLC‐HILIC system, detected with a stable microflow LC‐MS/MS system, and analyzed using the highly sensitive Glyco‐Decipher software equipped with a match‐between‐runs (MBR) quantification module. Proteins with ten or more dysregulated site‐specific glycans were selected by the protein‐centric strategy to identify biomarkers for diagnosis or staging.

To systemically screen proteins susceptible to aberrant glycosylation and further enhance the diagnostic performance for HCC, we designed a workflow by integrating the HRN platform with a protein‐centric screening strategy (Figure 1). A total of 200 clinical samples were separated into the discovery and validation cohorts by matching the clinical characteristics including age, gender and TNM (Tumor‐Node‐Metastasis) staging standard (Table S1). The discovery cohort consisted of 70 healthy controls (HCs) and 70 hepatocellular carcinoma patients (HCCs). The HCC cases were further categorized into 23 TNM‐I, 20 TNM‐II, 20 TNM‐III, and 7 TNM‐IV stages. The validation cohort included an independent group consisting of HCs (n = 30) and HCCs (n = 30), with HCCs further categorized into 9 TNM‐I, 9 TNM‐II, 9 TNM‐III, and 3 TNM‐IV stages. A HRN platform was applied to enable highly reproducible and sensitive analysis of the N‐glycoproteome in large cohorts of serum samples. In this platform, glycopeptide enrichment was performed using an automated HPLC‐HILIC strategy to avoid variability from manual operation; a stable microflow instead of nanoflow LC‐MS/MS system was used for glycopeptide analysis to enhance reproducibility; and the newly developed Glyco‐Decipher software with match‐between‐runs function was employed for N‐glycopeptide identification and quantification to improve sensitivity. Importantly, we proposed a protein‐centric strategy to prioritize the proteins susceptible to dysregulated glycosylation. And the alterations of dysregulated site‐specific glycans across different stages of HCC were characterized using Mfuzz analysis, aiming to identify distinct glycan structural features associated with disease progression. The diagnostic performance of individual site‐specific glycan from the proteins screened by the protein‐centric strategy was evaluated and compared with serum AFP. Finally, random forest models were constructed by randomly combining pairs of dysregulated site‐specific glycans from the discovery cohort to form biomarker panels, which were then tested and validated in an independent cohort. Based on the models' AUC performance, biomarker panels derived from proteins susceptible to dysregulated glycosylation were identified for HCC detection, particularly for early‐stage and AFP‐negative HCC.

### 2.2 Assessment of the Stability and Reproducibility of the Integrated Platform

Repeatability is essential for screening biomarker candidates, especially when measuring a large sample size over an extended experimental period. The LC‐MS/MS analysis of 200 individual samples in DDA mode through the microflow separation system required over ten days. Therefore, we designed a series of quality control samples (QCs) including batch‐QCs, interday‐MSQCs, and intraday‐MSQCs to assess the repeatability in sample preparation and LC‐MS/MS analysis. Both the batch‐QCs and MS‐QCs were derived from pooled serum N‐glycopeptide samples, and the MS‐QCs were analyzed in triplicate and each day to evaluate the intra‐day (within day) and inter‐day (different days) system stability. The workflow demonstrated robust performance in N‐glycoproteome identification and quantification across triplicates, inter‐day MS‐QCs, and batch‐QCs with relative standard deviations (RSD) below 6% and average Pearson correlation coefficients above 0.92 (Figure S1a–f), respectively.

The robust microflow system within the HRN platform ensured stable retention times even during long‐term and large‐scale sample analyses [22], thus benefiting MBR quantification. A clear visualization of MBR's powerful missing value imputation capability was demonstrated by comparing the quantified glycopeptides before and after the application of MBR using the QCs dataset (Figure S1g). Incorporating MBR‐based quantification reduced the proportion of site‐specific glycans with missing values from 88.5% to 38.1% and the proportion of quantitative values with missing values from 68.3% to 9.6% (Figure S1h), demonstrating that the MBR function in the newly developed Glyco‐Decipher software markedly improved data sensitivity. The enrichment specificities of QCs and individual samples (over 230 injections) averaged 93% (range: 88.7%–95.1%), indicating highly consistent glycopeptide enrichment (Figure S2). Overall, the robustness of the HRN platform ensured high data quality.

### 2.3 Match‐Between‐Run Quantification Enables In‐Depth Differential Analysis of Site‐Specific Glycans in HCC

To investigate glycosylation heterogeneity between healthy and HCC groups, and identify potential diagnostic biomarkers, we employed a “rectangular” biomarker strategy to perform glycoproteomic analysis on 200 clinical samples from the discovery and validation cohorts. In the discovery cohort, a total of 19,124 intact N‐glycopeptides and 18,430 site‐specific glycans were identified, covering 1,435 N‐glycans and 1,214 N‐glycosites across 820 glycoproteins. Comparative analysis of N‐glycoproteomes between HCC and HC groups revealed distinct glycosylation patterns, particularly elevated fucosylation (Figures S3, S4).

**FIGURE 2:**
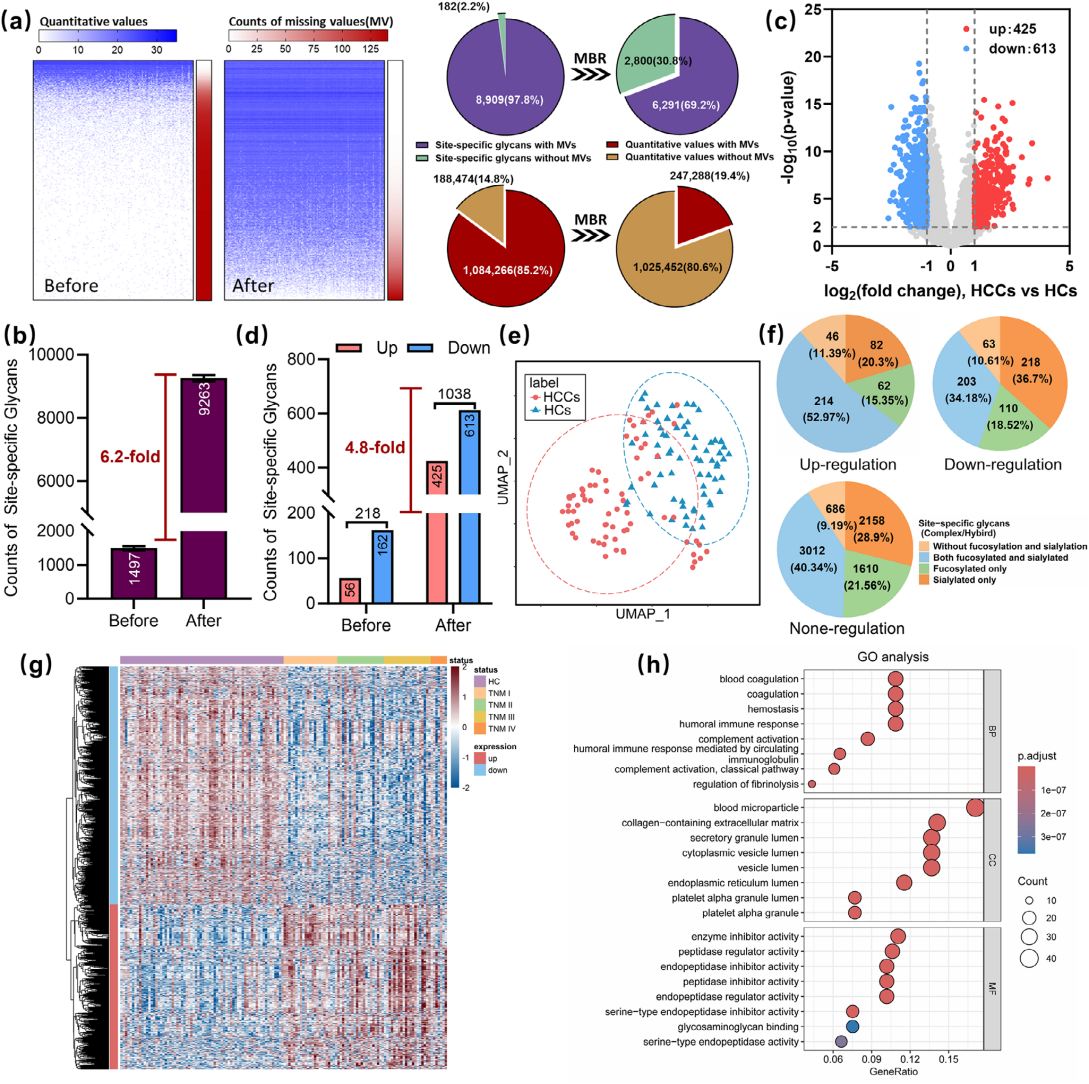
Quantitative analysis of site‐specific glycans in the discovery cohort. (a) Comparison of identification‐based method and label‐free quantification method (with match‐between‐runs scheme) in the discovery cohort dataset: (left) heatmap of quantitative values and the counts of missing values; (right) the proportion of missing values for site‐specific glycans and quantitative values. (b) The counts of quantifiable site‐specific glycans in the single‐shot profiling (before vs. after match‐between‐runs treatment). (c) Volcano plot comparing site‐specific glycans of HCCs versus HCs based on the discovery dataset (after‐MBR). (d) The counts of significantly dysregulated site‐specific glycans (before vs. after match‐between‐runs treatment). (e) Assessment of dysregulated site‐specific glycans for distinguishing HCCs from HCs using uniform manifold approximation and projection (UMAP). (f) Distribution of complex and hybrid glycoforms in the different regulated groups. (g) Heatmap depicting the abundance profiles of differentially abundant site‐specific glycans across five groups (HCs and TNM‐I, II, III, IV of HCCs). The quantitative data were log2‐normalized followed by z‐score data processing. (h) GO analysis of proteins with dysregulated site‐specific glycans in terms of biological processes (BP), cellular components (CC), and molecular functions (MF). The level of significance is represented by the color of the bar with scale, and the gene count is indicated by the diameter of the circle.

In quantitative glycoproteomics, we found the MBR dramatically decreased the ratio of quantified site‐specific glycans with missing values in the discovery dataset from 85.2% to 19.4% (Figure 2a). Strikingly, a 6.2‐fold increase in quantifiable site‐specific glycans was observed in single‐shot profiling after MBR application, enabling deep serum N‐glycoproteome quantitative analysis for subsequent investigation (Figure 2b). Compared to the QCs dataset, individual dataset had slightly more missing values in MBR‐quantification results due to the higher heterogeneity of the individual samples. Nonetheless, MBR still enabled the assignment of quantitative values to the vast majority of the data, providing a rich source for subsequent analysis. Statistical t‐test analysis between HCC and HC specimens in the discovery cohort yielded 1,038 dysregulated site‐specific glycans (p ≤ 0.01, absolute log2 fold change ≥ 1, Figure 2c), corresponding to 425 up‐regulated and 613 down‐regulated ones, respectively.  In contrast, t‐test analysis using the same criteria on the discovery cohort dataset before‐MBR only yielded 218 dysregulated site‐specific glycans (Figure S5a). Notably, MBR induced a 4.8‐fold increase in the number of significantly dysregulated site‐specific glycans, substantially enriching the candidates for diagnostic marker discovery (Figure 2d). It should be noted that Pan et al. identified 15,512 N‐linked intact glycopeptides (NIGPs) in 119 ovarian cancer tissues, yet only 351 (2.3%) exhibited quantifiable values across all samples, forcing their heterogeneity analysis to rely on this minimal subset [31]. Similarly, Liu et al. detected about 20,000 site‐specific glycans in 140 serum samples but retained merely 106 (0.5%) dysregulated glycopeptides after differential analysis for gastric cancer biomarker screening [22]. These studies underscore the critical bottleneck imposed by missing values in glycoproteomics. The MBR quantification method provides a way to enhance intact glycopeptide profiling and expand diagnostic marker candidates.

Uniform Manifold Approximation and Projection (UMAP) analysis of site‐specific glycans demonstrated a clear separation between the HCC and HC groups, with HCCs exhibiting a more dispersed data distribution, which indicates higher glycosylation heterogeneity (Figure 2e). In this study, the glycans were classified into three major categories based on their glycan structures: truncated glycans (Hex(<4)HexNAc(<3)Fuc(<2)), oligo‐mannose glycans (Hex(>3)HexNAc(2)Fuc(<2)), and complex/hybrid glycans. These complex/hybrid glycans were subdivided into four types based on glycan compositions: without fucosylation and sialylation, both fucosylated and sialylated, fucosylated‐only and sialylated‐only. Although the overall proportions of three major glycan categories remained similar across sample groups (Figure S5b), the striking compositional shift was found in the complex/hybrid glycan types. Specifically, glycans containing both fucosylation and sialylation (blue in Figure 2f) accounted for a significantly higher proportion (52.97%) in the up‐regulated group compared to the down‐regulated group (34.18%). In contrast, the sialylated‐only glycans (orange) showed a significantly reduced proportion in the up‐regulated group (20.30% vs. 36.70%), whereas the fucosylated‐only glycans (green) remained basically unchanged (Figure 2f). This suggested that disease onset may preferentially promote fucosylation of glycans already bearing sialic acid. In addition, the expression profiles of these dysregulated site‐specific glycans showed clear intergroup differences between HCC and HC samples (Figure 2g). Gene ontology (GO) enrichment analysis of the corresponding glycoproteins revealed many biological processes, notably hemostasis, immunity, and regulation of fibrinolysis (Figure 2h). Collectively, MBR enabled in‐depth identification and quantification of the serum site‐specific glycans in HCC, and highlighted the enhanced glycan fucosylation associated with the disease.

### 2.4 Screening of Proteins Susceptible to Dysregulated Glycosylation by a Protein‐Centric Strategy

**FIGURE 3:**
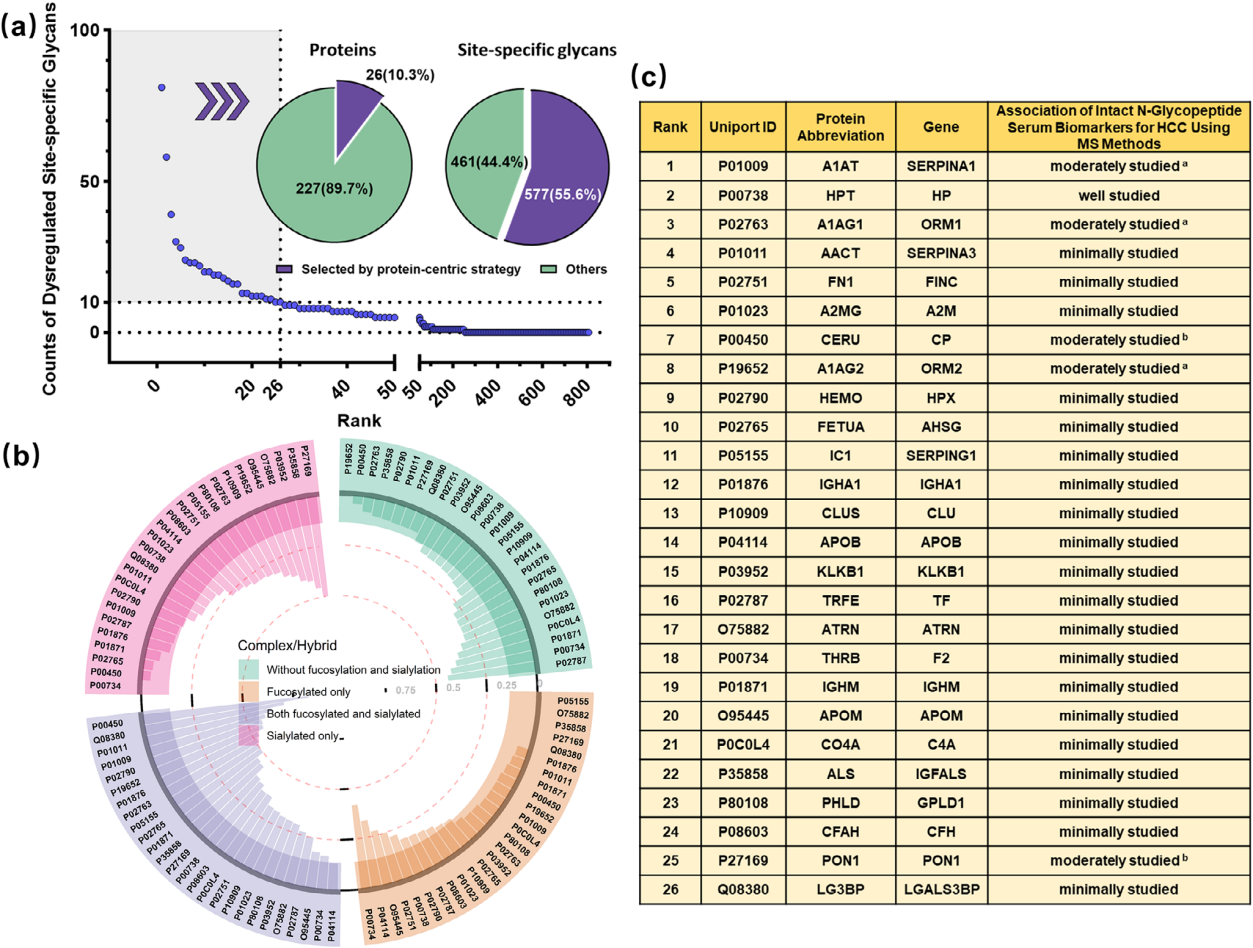
The proteins susceptible to dysregulated glycosylation screened by a protein‐centric strategy. (a) Distribution of dysregulated site‐specific glycans across proteins and prioritization of 26 proteins based on the protein‐centric strategy. (b) Distribution of complex and hybrid glycoforms among the dysregulated site‐specific glycans in the 26 prioritized proteins. (c) Based on the literature on the association of intact N‐glycopeptide serum biomarkers with HCC, the 26 prioritized proteins are categorized into three levels. Well studied: The protein has been purified from serum for focused analysis in many studies. Moderately studied^a)^: The protein has been purified from serum for focused analysis in one study. Moderately studied^b)^: Experiments were conducted on the whole serum level, and the glycopeptide of this protein was finally selected as biomarker. Minimally studied: Glycopeptides derived from the protein were identified as dysregulated, without focused analysis.

We next examined the distribution of dysregulated site‐specific glycans across proteins and found that proteins with a higher number of dysregulated site‐specific glycans were relatively rare, while those with fewer dysregulated site‐specific glycans were more abundant (Figure 3a). To uncover novel cancer‐associated proteins overlooked before and detect proteins susceptible to aberrant glycosylation, we proposed a protein‐centric strategy. This strategy involves ranking glycoproteins by their number of dysregulated site‐specific glycans and then selecting the top few as candidates susceptible to aberrant glycosylation. In this study, 253 glycoproteins corresponding to 1,038 dysregulated site‐specific glycans were identified in the discovery cohort. In order to identify a representative set of proteins while keeping the number manageable, the top 10% glycoproteins (n = 26), each containing more than 10 dysregulated site‐specific glycans, were selected for subsequent analysis (Table S2). Therefore, a threshold of ≥10 dysregulated site‐specific glycans per glycoprotein was applied to screen for proteins susceptible to aberrant glycosylation in subsequent analyses. Additionally, the 577 dysregulated site‐specific glycans carried by the 26 proteins (represented 10% of all glycoproteins with dysregulated site‐specific glycans) accounted for 55.6% of all dysregulated site‐specific glycans (Figure 3a), representing a broad and substantial coverage. Interestingly, almost all these proteins carried both up‐ and down‐regulated glycans (Figure S6a). Applying the same criteria to the validation cohort prioritized 21 proteins (Figure S6b), 19 of which overlapped with the discovery cohort (Figure S6c). The number of dysregulated site‐specific glycans per protein in both cohorts demonstrated good correlation (R^2^ = 0.81, Figure S6d). The high reproducibility result confirmed the robustness of the protein‐centric strategy. The protein names, abbreviations, and corresponding gene names were provided in the supplementary table (Tables S3, S4). We then systematically presented the glycan structures and glycosites corresponding to the dysregulated site‐specific glycans of 26 proteins screened from the discovery cohort (Figure S7, Table S5).

The glycan compositions of dysregulated site‐specific glycans across the 26 prioritized proteins were analyzed in the discovery cohort, focusing on the four subtypes of complex/hybrid glycans. A circular bar plot (Figure 3b) provided a comprehensive overview of proteins with pronounced aberrant fucosylation or sialylation. Notably, about 80% of the aberrant site‐specific glycans on P00450 (Ceruloplasmin) contained both fucosylation and sialylation, and ten proteins had over half of their dysregulated site‐specific glycans belonging to this subtype. In contrast, the other three glycan subtypes rarely exceeded 50% per protein, underscoring the central role of combined fucosylation and sialylation in disease progression.

Based on literature reports of intact N‐glycopeptides identified by MS‐based methods as serum biomarkers for HCC diagnosis, the proteins were categorized into three groups: well‐studied, moderately studied, and minimally studied (Figure 3c). In general, the protein‐centric strategy identified a subset of proteins previously well‐ or moderately‐studied in the literature. For example, P00738 (Haptoglobin) is a well‐studied glycoprotein as its aberrant glycosylation on purified protein from serum was explored for biomarker in many studies and P01009 (Alpha‐1‐antitrypsin) is a moderately‐studied glycoprotein as only one study reported the analysis with the purified protein from serum. The determination of these proteins validated the feasibility of the protein‐centric strategy. More importantly, the majority of the determined glycoproteins were minimally studied ones, as no focused analysis of those proteins was performed previously. Since multiple aberrant site‐specific glycans (≥10) were also observed in these glycoproteins, they represent promising candidates for further investigation in HCC biomarker discovery.

### 2.5 The Differential Site‐Specific Glycan Dataset Reveals Significant Alterations in N‐Glycosylation Associated with HCC Progression

**FIGURE 4:**
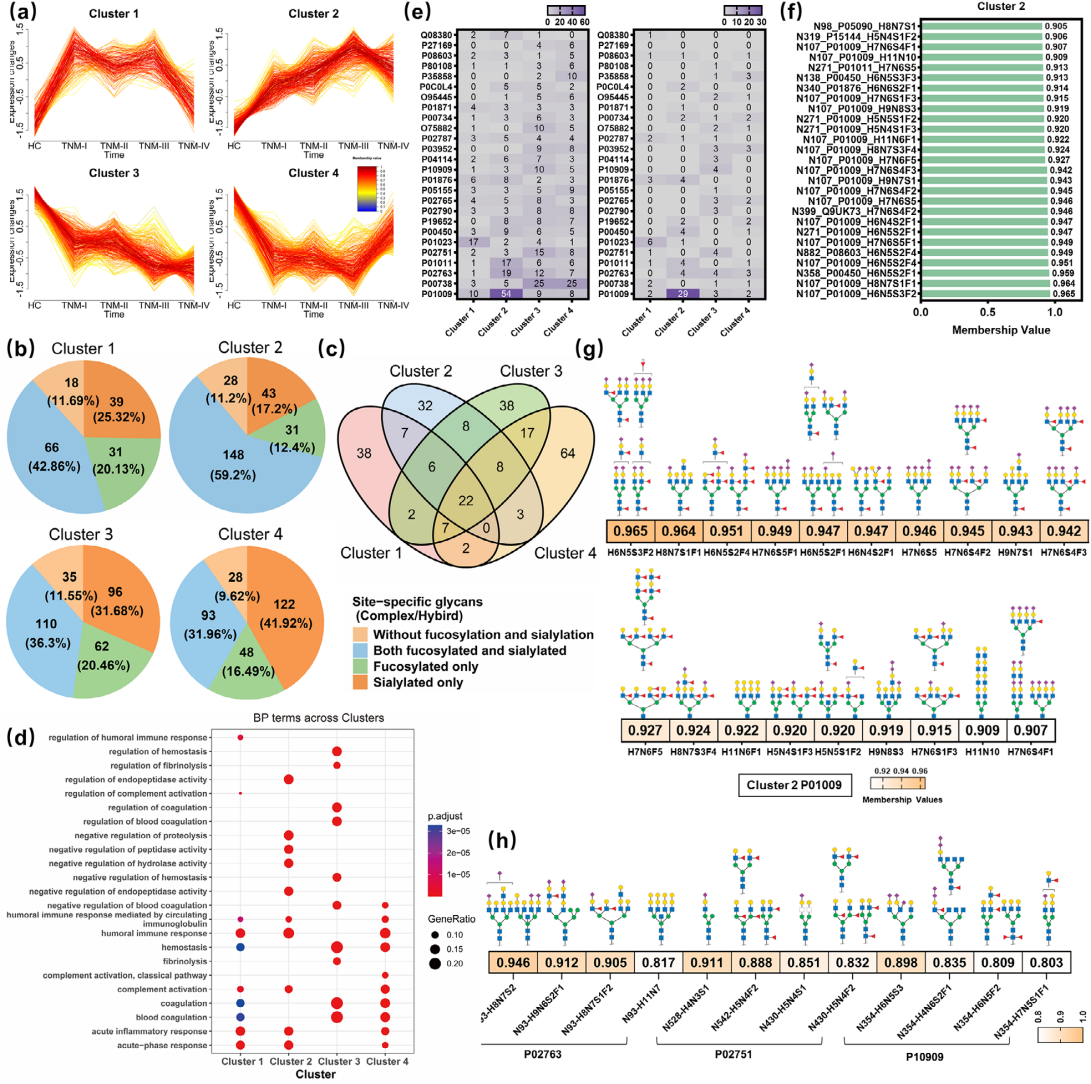
Clustering of dysregulated site‐specific glycans in the discovery cohort using the Mfuzz method. (a) Four clusters of dysregulated site‐specific glycans were identified with Mfuzz analysis. Among them, site‐specific glycans in Cluster 2 and Cluster 3 show a similar regulatory trend during the disease development. (b) Distribution of complex and hybrid glycoforms across the four clusters. (c) Venn diagram showing the overlap of proteins associated with the dysregulated site‐specific glycans across the four clusters. (d) GO analysis of proteins in terms of biological processes (BP) across four clusters. The level of significance is represented by the color of the bar with scale, and the gene ratio is indicated by the diameter of the circle. (e) Heatmap showing the frequency distribution of site‐specific glycans from the 26 prioritized proteins. The left panel displays all dysregulated glycans; the right panel displays those with a membership value > 0.8. (f) Ranking of site‐specific glycans with a membership value > 0.9 in Cluster 2. (g) The glycan structures of site‐specific glycans from a moderately‐studied protein P01009 (Alpha‐1‐antitrypsin) in Cluster 2 with the membership value > 0.9. (h) The glycan structures of site‐specific glycans from a moderately‐studied protein P02763 (Alpha‐1‐acid glycoprotein 1), and two minimally‐studied proteins P02751 (Fibronectin) and P10909 (Clusterin) in Cluster 3 with the membership value > 0.8.

To investigate glycosylation dynamics across HCC stages, the 1,038 dysregulated site‐specific glycans were grouped into 4 clusters using the Mfuzz method (Figure 4a), each displaying distinct abundance trends. Cluster 1 showed a rapid increase in stage I followed by fluctuations and a decline; Cluster 2 exhibited a gradual, basically monotonic increase; Cluster 3 demonstrated a progressive, basically monotonic decrease; and Cluster 4 declined sharply in stage I, then fluctuated and increased later. Notably, Cluster 2 and Cluster 3 exhibited consistent monotonic upward and downward trends, respectively, throughout disease onset and progression.

Then we analyzed the glycan compositions of complex or hybrid‐type site‐specific glycans across these four clusters (Figure 4b) and found that Cluster 1 and Cluster 2 were predominantly enriched in glycans carrying both fucosylation and sialylation, exhibiting significantly higher proportion of this subtype than Cluster 3 and Cluster 4, whereas Cluster 3 and Cluster 4 were enriched in sialylated‐only glycans. These findings were consistent with our previous analysis of complex/hybrid‐type glycans in different dysregulated categories (Figure 2f). We further explored the overlap of proteins associated with these site‐specific glycans across clusters. As shown in Figure 4c, the relatively low protein overlaps between any two clusters suggested that they were involved in different biological processes. GO analysis demonstrated that these variations reflected the dynamic biological processes associated with HCC, including immune responses, inflammation, and coagulation regulation (Figure 4d). Notably, the sharp changes in Cluster 1 and Cluster 4 during stage I suggested robust acute immune responses and alterations in coagulation, indicating the body's early attempts to counteract tumor growth. In contrast, the monotonic trends in Cluster 2 and Cluster 3 highlighted sustained immune modulation and fine‐tuned coagulation regulation during HCC progression, potentially associated with immune evasion, angiogenesis, and metastasis. Overall, these results illustrated the similar and distinct roles that proteins from different clusters play in regulating the biological processes underlying HCC pathogenesis and progression.

Assignment of a site‐specific glycan to a particular cluster was based on its highest membership value among the four clusters, reflecting the closest alignment with the cluster's expression dynamics. A membership threshold (>0.8) was applied to define high‐confidence cluster affiliations. Using this approach, 577 dysregulated site‐specific glycans from the 26 proteins prioritized by the protein‐centric strategy were assigned to the four clusters (Figure 4e, left), and the analysis was further refined by focusing on glycans with high‐confidence cluster affiliations (membership > 0.8, Figure 4e, right). Counts of these selected site‐specific glycans were presented in a heatmap, revealing that several proteins exhibited higher counts in Cluster 2 and Cluster 3. For instance, the site‐specific glycans on protein P01009 (Alpha‐1‐antitrypsin) were predominantly represented in Cluster 2, while proteins such as P02763 (Alpha‐1‐acid glycoprotein 1), P01011 (Alpha‐1‐antichymotrypsin), and P00450 (Ceruloplasmin) also showed notable enrichment and warrant further investigation. Interestingly, four site‐specific glycans with high‐confidence (>0.8) from P02763 with down‐regulation trend were also enriched in Cluster 3.

Then we investigated the glycan compositions and glycan structures for site‐specific glycans enriched in Cluster 2 with membership > 0.9 (Figure 4f), which consistently up‐regulated throughout HCC progression. Notably, site‐specific glycans with both sialylated and fucosylated modifications accounted for a large proportion (19/27), and particularly for the ones from a moderately‐studied protein P01009 (Alpha‐1‐antitrypsin). A closer investigation of their glycan structures indicated that they were predominantly multi‐branched and featured with both sialylated and core‐fucosylated modifications (Figure 4g). In contrast, the representative site‐specific glycans from a moderately‐studied protein P02763 (Alpha‐1‐acid glycoprotein 1), two minimally‐studied proteins P02751 (Fibronectin) and P10909 (Clusterin) in Cluster 3 exhibited less branching and displayed a lower degree of co‐occurrence between sialylation and fucosylation modifications (Figure 4h). In conclusion, distinct aberrant glycosylation patterns were observed on the moderately or minimally studied glycoproteins, highlighting that focused analysis of these proteins is required for discovering site‐specific glycan biomarkers.

### 2.6 Site‐Specific Glycans on Fibronectin Outperform AFP for Detecting Early HCC

**FIGURE 5:**
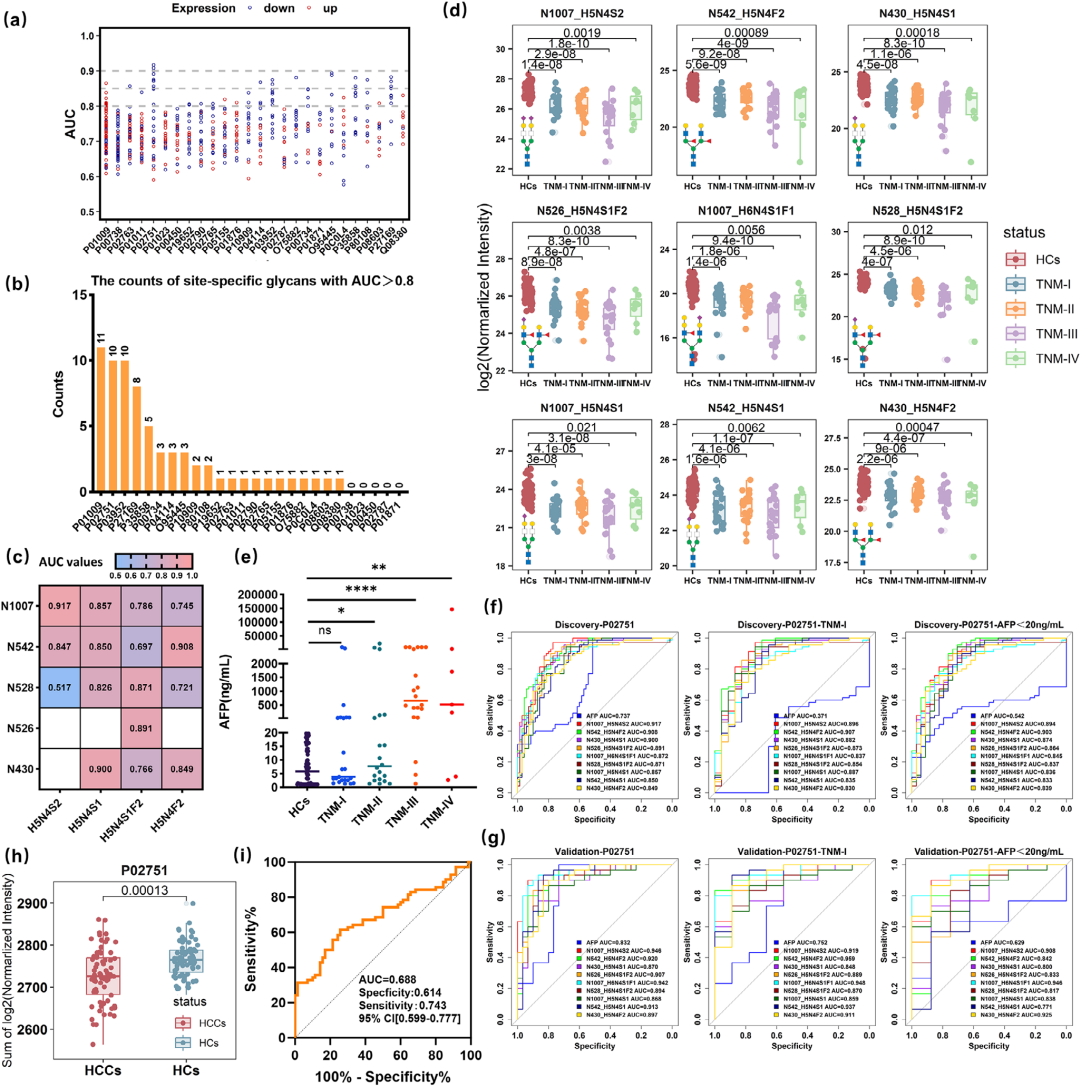
Site‐specific glycans on Fibronectin outperform AFP for detecting early HCC. (a) Scatter plot illustrating the distribution of AUC values for dysregulated site‐specific glycans from 26 proteins. Proteins are arranged left to right by decreasing the number of dysregulated site‐specific glycans. (b) Distribution of site‐specific glycans with AUC > 0.8 among those 26 proteins. (c) Heatmap of AUC values for site‐specific glycans from different glycosites and glycan compositions. (d) Abundance distribution of 9 candidate biomarkers with significant differences across different stages of HCC and the HC group. A two‐sided Wilcoxon test was employed to evaluate significance. Box plots show the median (center line), the 25th and 75th percentiles (box limits), and outliers. (e) Abundance distribution of serum AFP in the discovery cohort. ****, p value < 0.0001; ***, p value < 0.001; **, p value < 0.01; *, p value < 0.05; ns, p value > 0.05. (f) ROC curves of 9 candidate biomarkers from P02751 (Fibronectin) protein and serum AFP for distinguishing: HCCs (n = 70) vs. HCs (n = 70), TNM‐I HCCs (n = 23) vs. HCs (n = 70), and AFP‐negative HCCs (n = 34) vs. HCs (n = 70) in the discovery cohort. (g) ROC curves of 9 candidate biomarkers from P02751 (Fibronectin) protein and serum AFP for distinguishing: HCCs (n = 30) vs. HCs (n = 30), TNM‐I HCCs (n = 9) vs. HCs (n = 30), and AFP‐negative HCCs (n = 8) vs. HCs (n = 30) in the validation cohort. (h) Abundance of P02751 (Fibronectin) at the global‐glycosylation level (sum of all site‐specific glycan quantitation values) in the discovery cohort, showing a significant difference between HCC and HC groups. (i) ROC curve of P02751 (Fibronectin) protein at the global‐glycosylation level.

To uncover novel diagnostic markers beyond well‐characterized glycoproteins, we focused on site‐specific glycans (with an AUC greater than 0.8 for HCC detection) from the 26 glycoproteins in the discovery cohort (Figure 5a,b), and systematically dissected their glycan structures, abundance distributions, and diagnostic performance. By ranking the 26 proteins based on the number of site‐specific glycans with AUC above 0.8, we ultimately selected the top 5 proteins in the discovery cohort. Notably, the protein P01009 (Alpha‐1‐antitrypsin) exhibited 11 site‐specific glycans with diagnostic performance greater than 0.8, followed by P02751 (Fibronectin) and P03952 (Plasma kallikrein) with 10, P27169 (Serum paraoxonase/arylesterase 1) with 8, and P35858 (Insulin‐like growth factor‐binding protein complex acid labile subunit) with 5. The diagnostic performance of dysregulated site‐specific glycans from these five proteins was summarized in Table S6.

Among the top five proteins, P02751 (Fibronectin) is a minimally studied glycoprotein and no focused analysis has been reported previously for the screening of HCC serum glyco‐biomarkers. Dysregulated glycans such as H5N4S2, H5N4F2, H5N4S1F2 and H5N4S1 were found on multiple glycosites of Fibronectin. To evaluate the diagnostic performance (AUC) of these site‐specific glycans, we compared the AUC values of the 16 site‐specific glycans formed from the above four glycan compositions at five sites (N1007, N542, N528, N526, and N430). The results indicated that identical glycan compositions on different glycosites exhibited distinct AUC diagnostic performance (Figure 5c), highlighting the significance of site‐specific glycans as potential biomarkers for disease detection. In addition, 9 site‐specific glycans exhibited a diagnostic performance (AUC) exceeding 0.84 (Table S6), and their relative abundances across different stages of HCC and HC group were compared and illustrated in Figure 5d. Specifically, site‐specific glycans on P02751 showed statistically significant abundance differences between HCs and TNM‐I HCC cases (p < 0.01, Figure 5d), whereas the abundance of AFP did not differ significantly between the two groups (p > 0.05, Figure 5e). Next, we performed ROC analysis comparing AFP with the nine site‐specific glycans from P02751 (Fibronectin) protein in distinguishing HCs from all HCCs, TNM‐I HCCs, and AFP‐negative (AFP < 20 ng mL^−1^) HCCs. Based on AUC values, the nine site‐specific glycan candidates demonstrated superior diagnostic performance compared to serum AFP, even in the early‐stage or AFP‐negative samples (Figure 5f). This performance was further validated using an independent validation cohort (Figure 5g). In particular, the site‐specific glycan P02751_N1007_H5N4S2 demonstrated excellent and robust diagnostic performance across all settings, achieving AUC values of 0.917 and 0.946 for HCC diagnosis, 0.896 and 0.919 for early‐stage (stage I) HCC diagnosis, and notably, 0.894 and 0.908 for AFP‐negative HCC diagnosis in the discovery and validation cohorts, respectively.

Additionally, the diagnostic performance of site‐specific glycans from other minimally or moderately studied glycoproteins, P01009 (Alpha‐1‐antitrypsin), P03952 (Plasma kallikrein), P27169 (Serum paraoxonase/arylesterase 1) and P35858 (Insulin‐like growth factor‐binding protein complex acid labile subunit) was also investigated and shown in the Figure S8. Nonetheless, site‐specific glycans from Fibronectin still compared favorably to those from the other glycoproteins, particularly for detecting early‐stage or AFP‐negative cases. Moreover, when the total quantitation values of all site‐specific glycans on Fibronectin were aggregated to represent the overall glycosylation, a difference was observed between HCC and HC groups (Figure 5h). However, this difference was substantially smaller than the differences seen at the level of individual site‐specific glycans, resulting in limited performance for HCC detection (Figure 5i). Conclusively, site‐specific glycans on Fibronectin demonstrated superior performance compared to its overall glycosylation and serum AFP for HCC detection, particularly for the early‐stage (TNM‐I) and AFP‐negative cases.

### 2.7 The Combination of Site‐Specific Glycans from Two Glycoproteins Susceptible to Aberrant Glycosylation Enhances Diagnostic Performance

**FIGURE 6:**
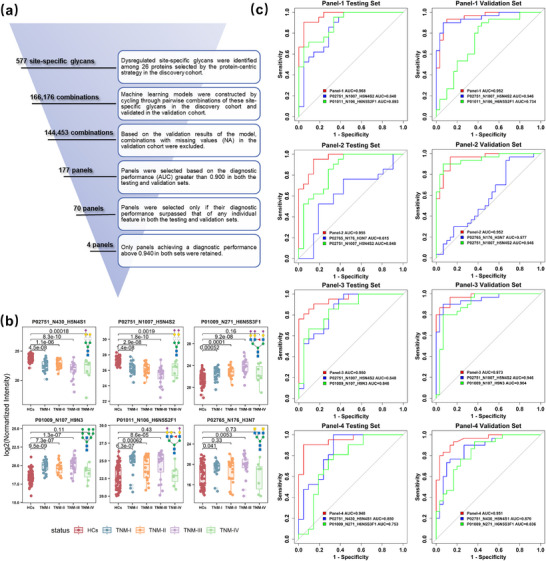
The combination of site‐specific glycans from two glycoproteins susceptible to aberrant glycosylation enhances diagnostic performance. (a) The procedure for selecting the four best‐performing panels. (b) The abundance distribution of six site‐specific glycans corresponding to the four panels. A two‐sided Wilcoxon test was employed to evaluate significance. Box plots show the median (center line), the 25th and 75th percentiles (box limits), and outliers. (c) ROC curves of four panels. Each panel comprises two site‐specific glycans identified by a machine learning model for complementary diagnostic performance. The panels were validated in a separate cohort (HCCs n = 30 vs. HCs n = 30) after initial evaluation in the discovery cohort (HCCs n = 70 vs. HCs n = 70).

Given that combinations of multiple biomarkers may enhance diagnostic performance, we next evaluated the discrimination power of panels composed of two site‐specific glycans for HCC diagnosis. To maximize feature coverage while accounting for potential synergistic effects between suboptimal individual markers, we included all dysregulated site‐specific glycans corresponding to the 26 glycoproteins susceptible to aberrant glycosylation in the initial machine learning model construction. Using a six‐tiered screening approach (Figure 6a), we first paired 577 dysregulated site‐specific glycans from the 26 proteins in the discovery cohort, generating 166,176 unique combinations. Machine learning models were iteratively trained to predict and validate the diagnostic performance of these combinations in distinguishing HCCs from HCs. After considering combinations present in both the discovery and validation cohorts, 144,453 combinations remained. Combinations were further filtered by AUC values > 0.900 in both cohorts, yielding 177 candidate panels. From these, we selected 70 panels whose diagnostic performance surpassed that of any single site‐specific glycan in both cohorts. Unexpectedly, each panel consisted of two site‐specific glycans from two different glycoproteins susceptible to aberrant glycosylation. Finally, four panels with AUC values greater than 0.940 in both the testing and validation sets were identified (Table S7), and their performance in early‐stage or AFP‐negative samples was further assessed.

Interestingly, 68 out of the 70 panels contained one up‐regulated site‐specific glycan and one down‐regulated site‐specific glycan in each panel (Table S8, given as a separate Excel file). Moreover, the four panels remained after the six‐tiered screening, and they also followed the same pattern (Figure 6b). As expected, the AUC performance of the selected four panels was consistently superior to individual features in distinguishing HCCs from HCs in both cohorts, even when one of the features had relatively lower diagnostic performance such as Panel‐2 (Figure 6c). Remarkably, these four panels maintained outstanding performance in both cohorts, achieving AUC values above 0.948 in the testing set and above 0.951 in the validation set (Figure 6c), thereby confirming their capacity for HCC diagnosis. Notably, Panel‐3, comprising P02751_N1007_H5N4S2 and the other site‐specific glycan P01009_N107_H9N3, demonstrated the best diagnostic performance, achieving AUC values of 0.950 and 0.973 for HCC diagnosis, 0.976 and 0.922 for early‐stage (stage I) HCC diagnosis, even 0.948 and 0.867 for AFP‐negative HCC diagnosis in the discovery and validation cohorts, respectively (Figure S9). These findings demonstrate that combining site‐specific glycans from distinct glycoproteins susceptible to aberrant glycosylation synergistically enhances diagnostic performance, even for early‐stage and AFP‐negative HCC.

## 3 Discussion

Hepatocellular carcinoma (HCC) remains a global health burden, with delayed diagnosis and limited sensitivity of current biomarkers such as alpha‐fetoprotein (AFP) driving high mortality rates. Aberrant glycosylation, a hallmark of cancer, has emerged as a rich source of diagnostic markers, yet conventional intact glycopeptide‐based glycoproteomic approaches have been constrained by small cohorts, limited identification and quantification capacity in single MS files, and only focusing on altered glycopeptides while overlooking their carrier proteins. This study addresses these gaps through a novel protein‐centric strategy integrated with advanced mass spectrometry (MS) workflows, yielding insights into HCC‐specific glycosylation dysregulation and identifying robust serum biomarker candidates. Our study advances HCC biomarker discovery through three critical innovations: initially, a Match‐Between‐Run (MBR) scheme was applied in a large‐scale HCC cohort study at site‐specific glycan level to advance single‐shot glycopeptide quantification and enhance the identification reproducibility and sensitivity; subsequently, we introduced a protein‐centric strategy to prioritize proteins with multiple dysregulated site‐specific glycans, aiming to discover proteins less‐studied by traditional approaches and characterize their dysregulated glycosylation patterns; finally, diagnostic panels combined with both up‐ and down‐regulated site‐specific glycans from prioritized proteins screened by the protein‐centric strategy were validated for their robust performance in detecting AFP‐negative and early‐stage cases.

Analyzing a large cohort of clinical specimens is a preliminary for discovering novel biomarker candidates with stable performance. However, the stochastic nature of DDA and the inherently low identification rate of glycopeptide spectra often lead to substantial missing values, resulting in the loss of valuable information. To address this, we integrated a “match between runs” (MBR) module into the Glyco‐Decipher [29] to identify shared glycopeptides across files and impute missing values. This technical breakthrough enabled robust analysis of 200 serum samples (100 HCC cases and 100 controls). The result indicated that this approach successfully reduced the missing value proportion in the discovery cohort from 85.2% to 19.4%, and a 6.2‐fold increase in quantifiable site‐specific glycans was observed in single‐shot profiling after MBR application, enabling deep serum N‐glycoproteome quantitative analysis for subsequent investigation. Consequently, 1,038 dysregulated site‐specific glycans (p ≤ 0.01, absolute log2 fold change ≥ 1) were detected in the discovery cohort, resulting in a 4.8‐fold increase compared to conventional method, substantially enriching the candidates for diagnostic marker discovery.

The protein‐centric strategy prioritized 26 glycoproteins susceptible to aberrant glycosylation. And these dysregulated site‐specific glycans accounted for 55.6% of the total dysregulated site‐specific glycans, highlighting the comprehensive data coverage provided by this strategy. These dysregulated site‐specific glycans demonstrated distinct glycosylation patterns in the up‐regulated and down‐regulated groups. Glycoforms that were multi‐branched and featured both sialylated and core‐fucosylated modifications dominated in the up‐regulated group. Conversely, the down‐regulated group exhibited glycoforms with fewer branches and a lower degree of co‐occurrence between sialylation and fucosylation modifications. Investigation on the complex/hybrid glycans between the up‐regulated and the down‐regulated groups implied that the onset and progression of HCC may preferentially promote fucosylation of glycans already bearing sialic acid, highlighting the enhanced glycan fucosylation associated with the disease.

Among the identified 26 proteins susceptible to aberrant glycosylation, a subset has been purified from HCC serum samples for the screening of glycopeptide biomarkers via LC‐MS/MS [14, 15, 16, 17, 18, 19, 32]. For example, Haptoglobin (P00738) is a well‐studied protein in HCC. While methodological differences exist between single‐protein enrichment and global serum glycoproteomics, our findings show both similarities and distinctions compared to prior work. First, the diagnostic performance of site‐specific glycans on protein P00738 was suboptimal. In our dataset, all 58 dysregulated site‐specific glycans on P00738 exhibited AUC values below 0.800 for HCC diagnosis and were not selected for detailed evaluation. This observation aligns with findings in the literature [17], where glycoproteomic analysis of purified haptoglobin from serum also reported moderate diagnostic power (highest AUC = 0.800 for the glycoform N241_A3G3F1S3) in discriminating cirrhosis from HCC. Secondly, purifying individual proteins from serum enables targeted analysis, eliminates interference from high‐abundance proteins, and thereby facilitates the detection of low‐abundance glycopeptides. This focused approach allows for resolving glycopeptide isomers and identifying potential isomer biomarkers, as demonstrated for Haptoglobin (P00738) [14] and Alpha‐1‐antitrypsin (P01009) [32]. It is worth noting that owing to our larger sample cohort (n = 200), the high‐sensitivity spectral interpretation enabled by the Glyco‐Decipher software, and the application of the MBR strategy for imputing missing values in glycopeptide quantification, our study achieves more comprehensive glycopeptide identification at the individual protein level. Furthermore, by conducting a global serum‐level analysis and employing a protein‐centric strategy, this study profiles a broad range of proteins and thus identifies new candidates with superior diagnostic performance. Excitingly, Fibronectin (P02751), a minimally‐studied glycoprotein, emerged as the top candidate. Fibronectin is a multifunctional glycoprotein distributed in the extracellular matrix (ECM) and body fluids, and it mediates cell adhesion and migration by binding to integrins and other ECM components, contributing to pathological processes by regulating cell signaling and ECM remodeling [33]. The soluble dimeric form of fibronectin from body fluids is secreted by hepatocytes. In our analysis of fibronectin, nine down‐regulated site‐specific glycans demonstrated AUC values exceeding 0.840. All these glycoforms are simple bianternary structures, four are modified only with sialylation, two are modified only with fucosylation, and three possess both sialylation and fucosylation. This suggests that alterations in sialylation or fucosylation of fibronectin may indicate changes in hepatic synthetic function. Herein, its site‐specific glycan P02751_N1007_H5N4S2 exhibited exceptional diagnostic performance in detecting HCC with AUC values of 0.917 and 0.946 in the discovery and validation cohorts, respectively, significantly outperforming serum AFP. Moreover, it also demonstrated robust performance in detecting early‐stage (TNM‐I) (AUC: 0.896) and AFP‐negative HCC cases (AUC: 0.894), whereas serum AFP lost discrimination power with AUC values of 0.371 and 0.542.

To maximize feature coverage for detecting HCC, machine learning models were constructed using paired dysregulated site‐specific glycans from the 26 proteins. After a six‐tiered filtration, four panels that demonstrated superior performance in HCC diagnosis (AUC > 0.940 in both cohorts) were selected, each containing one up‐regulated and one down‐regulated site‐specific glycan. Notably, the combination of aforementioned site‐specific glycan P02751_N1007_H5N4S2 on Fibronectin with P01009_N107_H9N3 on Alpha‐1‐antitrypsin yielded enhanced performance with AUC values of 0.950/0.973 for HCC diagnosis, 0.976/0.922 for detecting TNM‐I HCC, and 0.948/0.867 for AFP‐negative HCC cases detection in the discovery and validation cohorts, respectively. A new HCC serum biomarker, PIVKA‐II/DCP [34], has been used either in combination with AFP [35] or integrated into multi‐factors scoring systems like GALAD [36] /GAAD [37] for more effective HCC surveillance. Studies have shown that DCP achieves a diagnostic AUC of 0.790 in distinguishing AFP‐negative (AFP < 20 ng mL^−1^) HBV‐HCC from CHB [38], and an AUC of 0.843 in distinguishing HCC from non‐HCC conditions [35]. Since PIVKA‐II/DCP and our identified glycoprotein biomarkers are involved in distinct biological pathways in hepatocarcinogenesis, the combination of these biomarkers has the potential to achieve improved performance in detecting early HCC or AFP‐negative cases and merits exploration in future studies.

In summary, by integrating a protein‐centric strategy with MBR‐enhanced glycoproteomics, this study enabled the detection of low‐abundance glycopeptides previously excluded from analysis, identified robust HCC biomarkers and provided a framework for prioritizing glycoproteins susceptible to aberrant glycosylation in cancer. The findings highlight the potential of site‐specific glycans as superior diagnostic tools, particularly for addressing the critical challenge of detecting AFP‐negative and early‐stage HCC subsets. However, it should be noted that this study has several limitations. First, as a discovery‐phase investigation, we identified a novel panel of site‐specific glycan biomarkers. To translate these candidates toward clinical application, future studies should focus on developing high‐throughput assays, such as ELISA or targeted mass spectrometry approaches (e.g., multiple reaction monitoring or parallel reaction monitoring), and conducting multi‐center validation in diverse patient cohorts [39]. Second, given that patients with cirrhosis face an annual HCC risk of 2%–8%, primarily driven by chronic hepatitis B (HBV) and other causes of cirrhosis (e.g., hepatitis C [HCV]) [39], the inclusion of samples from patients with hepatitis or cirrhosis would enable a more comprehensive characterization of glycoprotein changes preceding HCC and identify novel site‐specific glycan biomarkers distinguishing cirrhosis from HCC. Third, our dataset provided quantitative information only at the glycopeptide level, lacking corresponding protein‐level quantification. And the integration of corresponding protein‐level quantitative data will provide a more precise and complete picture of site‐specific glycosylation dynamics in hepatocarcinogenesis, which merits investigation in future studies. Collectively, this study established a foundation for advancing glycoproteomic research from discovery to clinical application.

## 4 Experimental Section

### 4.1 Chemicals and Reagents

Urea, NH_4_HCO_3_, dithiothreitol (DTT), iodoacetamide (IAA), trifluoroacetic acid (TFA), formic acid (FA), and trypsin (bovine, TPCK‐treated) were purchased from Sigma (St. Louis, MO, USA). Acetonitrile (ACN, HPLC grade) was purchased from Merck (Darmstadt, Germany). The deionized water was obtained from the Wahaha Group Co. Ltd.

### 4.2 Clinical Sample Collection

The study protocol was approved by the Ethics Committee of the Second Hospital of the Dalian Medical University, Da Lian, China. Written informed consent was obtained from all participants. The study design and conduct complied with all relevant regulations regarding the use of human study participants and was conducted in accordance with the criteria set by the Declaration of Helsinki. All clinical serum samples were collected in the bio‐sample bank of the Second Hospital of the Dalian Medical University. HCC patients and healthy controls in two cohorts (discovery and validation cohorts) were well‐matched for gender and age. The clinical characteristics were summarized in Table S1.

### 4.3 Intact N‐Glycopeptide Enrichment in the HRN Platform

10 µL of individual human serum from HCC patients, healthy controls, or QC samples were diluted ten times with 8 M urea/0.1 M NH_4_HCO_3_, and then denatured by reduction (with 20 mM DTT at 37°C for 2 h) followed by alkylation (with 40 mM IAA at 25°C for 40 min). After urea concentration of the mixture was reduced to below 2 M with 0.1 M NH_4_HCO_3_, protein digestion was carried out with trypsin at an enzyme‐to‐protein ratio of 1:20 (w/w) at 37°C for 17 h. Tryptic peptides were subsequently desalted using Oasis HLB C18 cartridges (Waters) and then lyophilized. Glycopeptide enrichment and MS analysis of intra‐batch samples were performed randomly to avoid bias. Intact N‐glycopeptides were enriched by the automated N‐glycopeptide enrichment method, as previously reported [40]. The only difference was that this experiment used one column of the same material with different specifications (100 mm × 4.6 mm I.D.). In brief, the lyophilized tryptic peptides were redissolved in 0.1% TFA/75% ACN and injected automatically onto a HILIC column at a flow rate of 900 µL min^−1^. The glycopeptide fraction was collected after the non‐glycopeptide fraction was washed away. The following sample could be injected after system washing and re‐equilibration. The whole enrichment cycle for each sample needs 20 min.

### 4.4 Microflow LC‐MS/MS Analysis in the HRN Platform

10 µg glycopeptides were resuspended in 0.1% FA and submitted for microflow LC‐MS/MS analysis, which was performed in an Ultimate 3000 LC system coupled online to an Orbitrap Exploris 480 mass spectrometer (Thermo Fisher Scientific, USA). Glycopeptides were separated on a 1 mm × 15 cm ACQUITY UPLC Peptide CSH C18 column (130 Å, 1.7 µm; Waters), heated to 45°C, with a flow rate of 50 µL min^−1^. The following gradient was applied: 0–0.5 min, 4%–4% B; 0.5–1 min,4%–9% B; 1–52 min, 9%‐45% B; 52–54 min, 45%–95% B; 54–58 min, 95%–95% B; 58–58 min, 95%‐4% B; 58–60 min, 4%–4% B. For shotgun glycoproteomics, DDA mode was operated to switch between MS and MS/MS acquisition. Full scan MS spectra were collected from 350 to 1800 m/z at a resolution of 60 000, with a normalized AGC target of 300 and a maximum injection time (IT) of 25 ms. MS/MS scans were performed at a resolution of 30 000 using an isolation window of 2 m/z, with a normalized AGC target of 200 and a maximum IT of 100 ms. Glycopeptide fragmentation was performed by stepped HCD with normalized energy of 20%, 30% and 40%.

### 4.5 Data Analysis and Preprocessing

For N‐glycopeptide identification, raw data were searched with Glyco‐Decipher software [30] against the human UniProt database (20350 protein entries released in 2021_06). The spectra were searched using precursor and fragment ion tolerance of 10 and 20 ppm, respectively. The search was restricted to tryptic peptides allowing up to three missed cleavages. Cysteine carbamidomethylation (C +57.022 Da) was specified as a fixed modification. Methionine oxidation (M +15.995 Da) was set as a variable modification. False discovery rate (FDR) of glycopeptide spectrum match (GPSM) was restricted to less than 1%. And then, the.gpid files were sent to the GlyPep‐Quant quantification software to perform match‐between‐run analysis. And the details of MBR implementation workflow and score thresholds applied to control false positive rates can be found in the reference [29]. Profiles from the 200 samples of the discovery and validation cohorts were involved in the mutual matching process. The data were split into two parts based on the file names of the discovery and validation cohorts, forming the initial file for the quantitative analysis. Notably, the results of Glyco‐Decipher contain two types of glycopeptides: one type includes glycopeptides with glycans from the GlyTouCan database, and the other includes glycopeptides with modified glycans identified by the monosaccharide stepping method. Unless otherwise stated, the identification results from Glyco‐Decipher contain both types of glycopeptides, while the quantification results include only those with glycans from the GlyTouCan database.

Site‐specific glycans with 70% valid values within at least one group were retained. After filtering, 8,902 site‐specific glycans remained in the discovery cohort, and 8,893 site‐specific glycans remained in the validation cohort. Afterward, median normalization was applied to the dataset, followed by log2 transformation and normal distribution interpolation, resulting in the data‐processed quantitative file. All subsequent data analyses were conducted based on this pre‐processed file. And then the Student's t‐test was performed for HCC patients and healthy controls using GP‐Marker [41] software. Dysregulated site‐specific glycans were determined using the standard of p‐value ≤ 0.01 and absolute log2 fold change ≥ 1. And the random forest models constructed iteratively were configured with 500 trees and a mtry parameter of 1. During the construction of the machine learning model, the discovery cohort data (containing 140 samples) were first normalized using the z‐score method. These samples were then randomly split into train‐set and testing‐set at a 7:3 ratio, forming two independent datasets. The train‐set was used for model construction, while the testing‐set was used for model testing. Subsequently, the validation cohort data were normalized independently and used for model validation. To evaluate model stability and significance, we performed a 5‐fold cross‐validation (mtry = 1, 500 trees) using the R caret package and conducted 1,000 permutation tests to generate a null distribution, calculating the resultant statistical significance (p‐value) of the model performance. Detailed methodologies and the corresponding results were provided in the Supporting Information (Figure S10 and Table S9). The long names of monosaccharides were abbreviated to single‐letter codes (H, Hex; N, HexNAc; S, NeuAc; F, Fuc). The glycan structures were sourced from the GlyTouCan database. Notably, blue indicates that the glycan unit has been confirmed as GlcNAc. White indicates that the glycan unit was identified only as HexNAc, without specific discrimination between GlcNAc and GalNAc. The figures in the article were generated using either R programming or GraphPad Prism.

### 4.6 Data  Availability

All raw data and search results were uploaded onto the jPOST repository [42]. The accession numbers were JPST003880 for JPOST and PXD065283 for ProteomeXchange.

## Author Contributions

M.D., D.S., and M.Y. conceived the project. L.L. performed the experiments and analyzed the results. T.M. and J.M. enrolled patients and collected samples. L.L., T.M., and Y.L. subpackaged the clinical samples. Z.G. and X.L. provided HILIC columns. H.Z. and Z.F. developed the new version of Glyco‐Decipher. Q.L. and J.Z. provided code assistance. T.Y. and Y.W. maintained the mass spectrometry. L.L., Q.L., Z.F., Y.W., X.L., M.D. and M.Y. discussed the results. L.L., M.D., and M.Y. wrote the manuscript.

## Conflicts of Interest

The authors declare no conflicts of interest.

## Supporting information




**Supporting File**: advs74274‐sup‐0001‐SuppMat.docx.

**Supporting File**: advs74274‐sup‐0002‐SuppMat.xlsx.

**Supporting File**: advs74274‐sup‐0003‐SuppMatS8.xlsx.

## Data Availability

The data that support the findings of this study are available in the supplementary material of this article.
