# Supplementary material for: A Protein‐Centric Strategy Coupled with Match‐Between‐Run Glycoproteomics Enables Discovery of Robust Site‐Specific Glycan Biomarkers for Hepatocellular Carcinoma
Source: Adv Sci (Weinh). 2026 Feb 12;13(22):e16299. doi: 10.1002/advs.202516299 (PMC13088319; doi:10.1002/advs.202516299)
Supplement: Supplementary file 1 — Supporting File: advs74274‐sup‐0001‐SuppMat.docx. [file ADVS-13-e16299-s003.docx]

Supporting Information

**A Protein-Centric Strategy Coupled with Match-Between-Run Glycoproteomics Enables Discovery of Robust Site-Specific Glycan Biomarkers for Hepatocellular Carcinoma**

*Lei Liu, Taiheng Ma, Qi Liu, He Zhu, Zheng Fang, Jiahong Ma, Ting Yu, Yan Wang, Jiahua Zhou, Xiaoyan Liu, Yaqian Li, Zhimou Guo, Xinmiao Liang, Mingming Dong*, Deguang Sun* and Mingliang Ye**

Lei Liu and Taiheng Ma contributed equally in this work.

Lei Liu ^1,2^, Qi Liu^1,2^, He Zhu^1^, Zheng Fang^1^, Ting Yu^1,2^, Yan Wang^1^, Jiahua Zhou^1,2^, Xiaoyan Liu^1^, Zhimou Guo^1^, Xinmiao Liang^1^, and Mingliang Ye^1,2^

Address^1^: State Key Laboratory of Medical Proteomics, CAS Key Laboratory of Separation Science for Analytical Chemistry, Dalian Institute of Chemical Physics, Chinese Academy of Sciences, Dalian, 116023, China

Address^2^: University of Chinese Academy of Sciences, Beijing, 101408, China

E-mail: mingliang@dicp.ac.cn

Taiheng Ma, Jiahong Ma, Deguang Sun

Address: Division of Hepatobiliary and Pancreatic Surgery, Department of General Surgery

The Second Hospital of Dalian Medical University, Dalian 116021, China

E-mail: sdgdoctor@dmu.edu.cn

Yaqian Li, Mingming Dong

Address: MOE Key Laboratory of Bio-Intelligent Manufacturing School of Bioengineering, Dalian University of Technology, Dalian 116000, China

E-mail: dongmm@dlut.edu.cn

**Table of Contents:**

1. Figure S1-S10

2. Table S1-S7, S9

3. Table S8 is given as a separate file (excel)

4. Supplementary data is given as a separate file (excel)


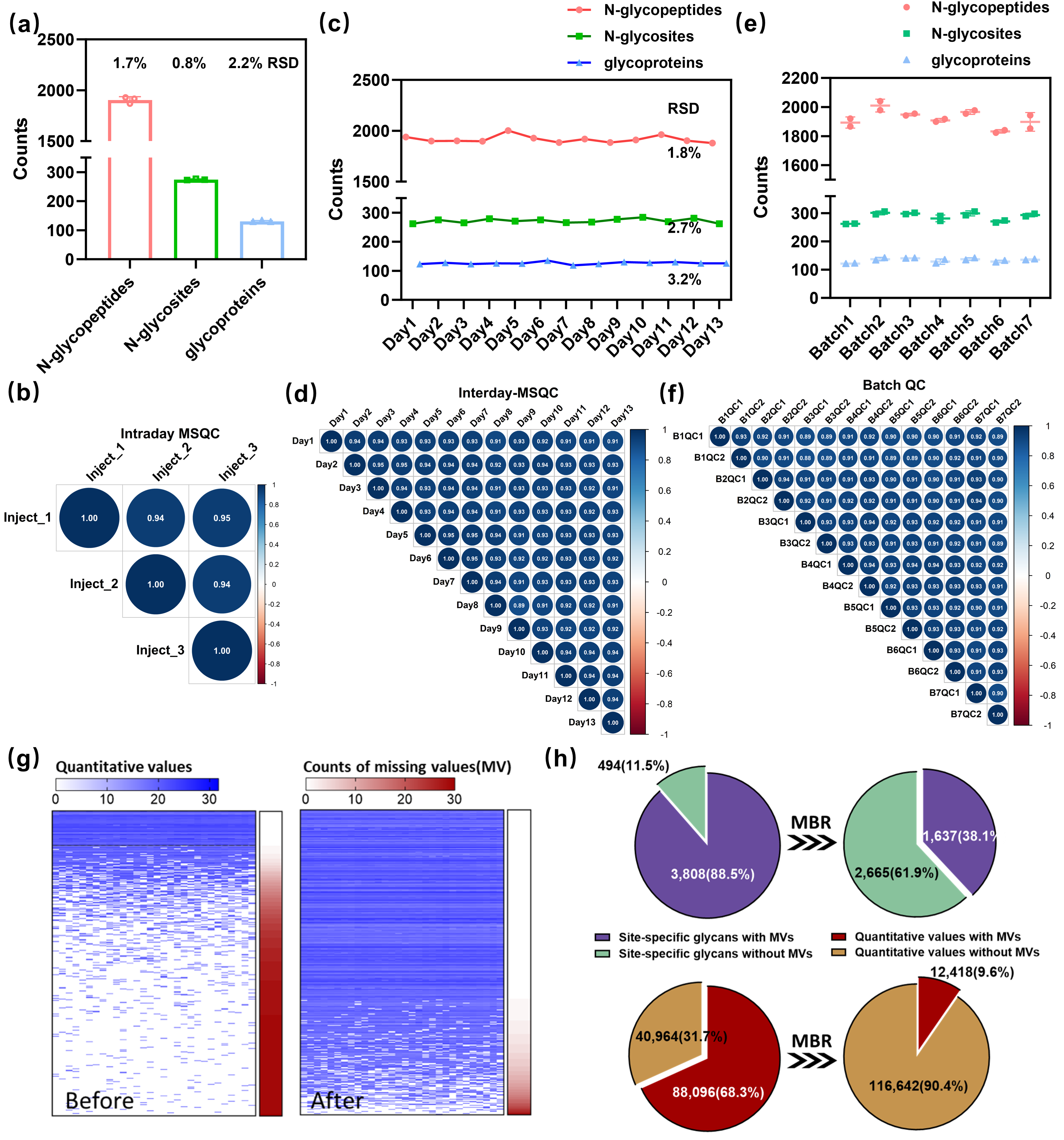


**Figure S1 Performance assessment of the HRN platform based on quality control samples (QCs) analysis.** (a, c, e) The number of N-glycopeptides, N-glycosites and glycoproteins identified from three intraday-MSQCs (a), thirteen MS-QCs with one MS-QC per day for thirteen days (c) and fourteen batch-QCs with two batch-QCs per batch for a total of seven batches (e). (b, d, f) Pearson correlation coefficients of quantified site-specific glycans of intraday-MSQC (b), interday-MSQC (d) and batch QC (f). (g) Heatmap of quantitative values and the counts of missing values (MV) in the QCs dataset using method based on identification results or label-free quantification method with match-between-runs. (h) The proportion of missing values for site-specific glycans and quantitative values in the QCs dataset using method based on identification results or label-free quantification method with match-between-runs.

Above data indicated that the workflow has robust performance in N-glycoproteome identifications across triplicates (Figure S1a) and inter-day MS-QCs, enables the identifcation of around 1,900 intact N-glycopeptides, 270 N-glycosites, and 120 glycoproteins, all with RSDs below 4% (Figure S1c). In addition, both the intra-day and inter-day analysis demonstrated high reproducibility in glycopeptide quantification, with average Pearson correlation coefficients of 0.945 and 0.929, respectively (Figure S1b and S1d). The stability of mass spectrometry runs over ten consecutive days was comparable to the triplicate sample injections, demonstrating that the microflow LC-MS/MS system maintained a high robustness when processing large-scale samples. The 200 samples from the discovery and validation cohorts were divided into seven batches for sample preparation, and two batch-QC samples were added to each batch for evaluating the parallelism of experimental operations. Thus, the discovery cohort included 28 samples in each batch, and the validation cohort consisted of 30 samples in each batch. Batch-QCs were prepared by evenly dividing pooled serum and processed alongside individual samples during the experiment. The RSDs for the number of N-glycopeptides, glycosites, and glycoproteins identified from fourteen batch-QCs were all below 6% (Figure S1e), and the average Pearson correlation coefficient for quantified N-glycopeptides of batch-QCs was 0.915 (Figure S1f), indicating the high reproducibility of the whole sample preparation process.


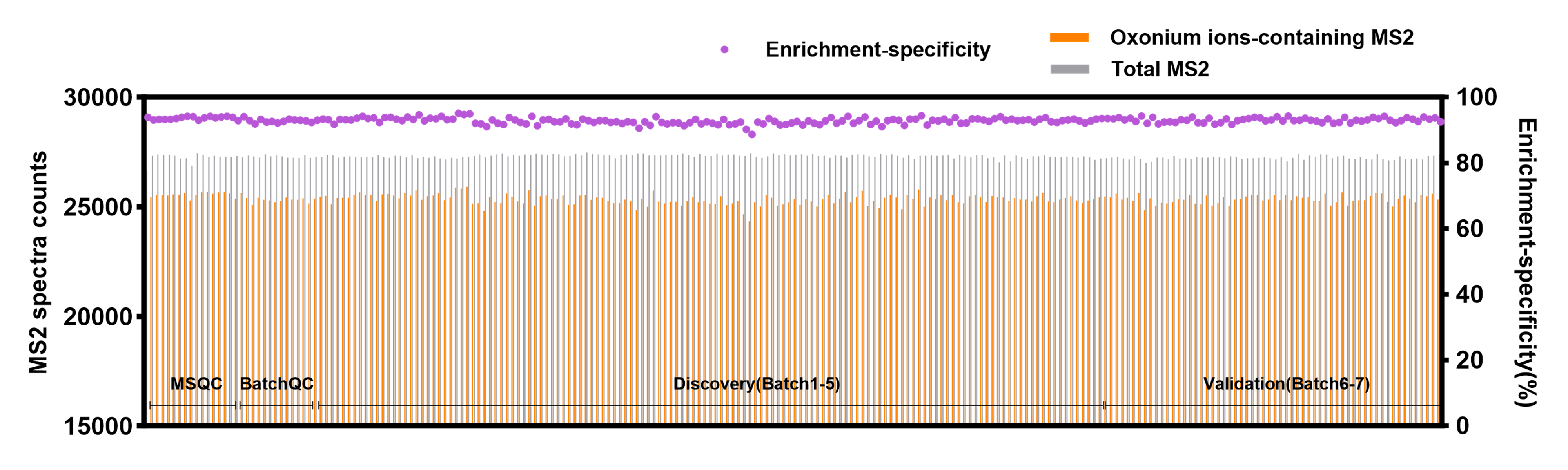


**Figure S2 Enrichment specificity of the HRN platform.** The enrichment specificity (dot plot) and MS2 spectra counts (bar plot) were analyzed for sixteen MS-QCs, fourteen batch-QCs, and all 200 participants across the discovery and validation cohorts. Enrichment specificity was determined by calculating the ratio of oxonium ion-containing spectra to the total MS2 spectra detected using Glyco-Decipher.


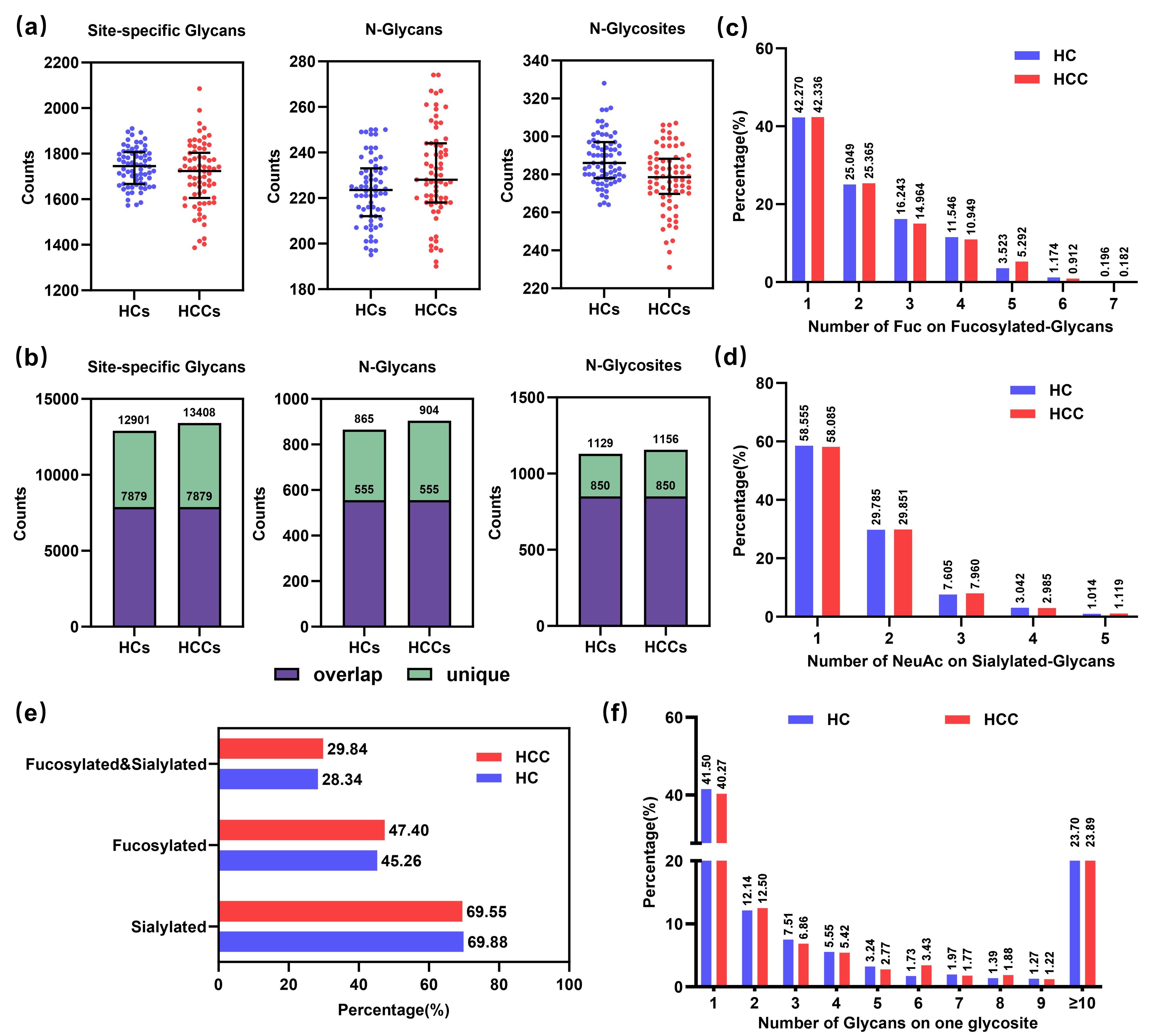


**Figure S3 Serum N-glycoproteome analysis based on the identification results in the discovery cohort.** (a) The number of site-specific glycans, N-glycans, and N-glycosites identified from individual samples. (b) The distribution of site-specific glycans, N-glycans, and N-glycosites identified from HCC group and HC group. (c-d) Distribution of Fuc-units on fucosylated-glycans (c) and NeuAc-units on sialylated-glycans (d) identified from HCC group and HC group. (e) Distribution of the percentage of fucosylated and sialylated glycans, sialylated glycans, and fucosylated glycans. (f) Distribution of the different numbers of glycans on one glycosite.


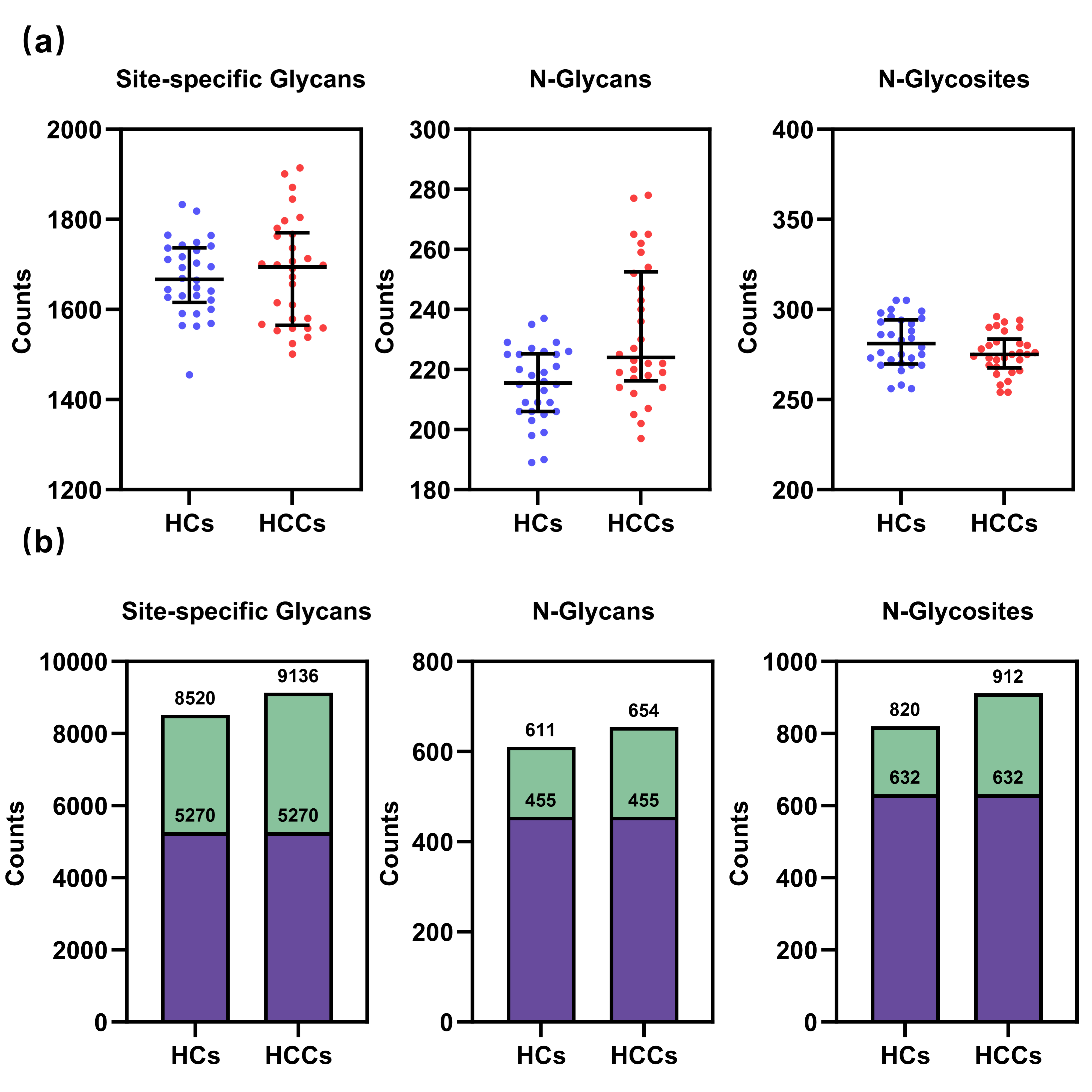


**Figure S4 Serum N-glycoproteome analysis based on the identification results in the validation cohort.** (a) The number of site-specific glycans, N-glycans, and N-glycosites identified from individual samples. (b) The distribution of site-specific glycans, N-glycans, and N-glycosites identified from HCC group and HC group.

Comparing the N-glycoproteome identifications, HCCs exhibited greater variability than HCs in the counts of site-specific glycans, N-glycans and N-glycosites (Figure S3a), along with a slightly higher number of unique identifications across all three levels (Figure S3b). We also observed similar data distribution in the validation cohort, while the variability of N-glycosites was comparable between the two groups (Figure S4). Next, we categorized the site-specific glycans into sialylated glycans, fucosylated glycans, and glycans containing both fucosylation and sialylation based on the presence of fucose and sialic acid units. Although the percentages of different numbers of sialic acid and fucose units in sialylated and fucosylated glycans were basically comparable between the HCC and HC groups (Figure S3c, d), the fucosylated glycans containing five fucose units showed a 5.292% proportion in HCCs, slightly higher than the 3.523% in HCs. Similarly, the proportion of sialylated glycans in HCCs was comparable to that of HCs, whereas the fucosylated glycans and glycans containing fucosylation and sialylation occupied a marginally higher proportion in HCCs (Figure S3e). These results were consistent with the observed increase in fucosylation levels in HCC as mentioned in other literature.^[1–3]^ Using the HRN platform's capability to localize glycosylations to specific glycosites, we discovered that in both HCCs and HCs, approximately 60% of the glycosites were modified by more than one N-linked glycan (Figure S3f), demonstrating the prevalence of glycosylation. Therefore, the distinct glycosylation patterns observed at the identification level strongly drive the need for in-depth quantitative glycoproteome analysis.


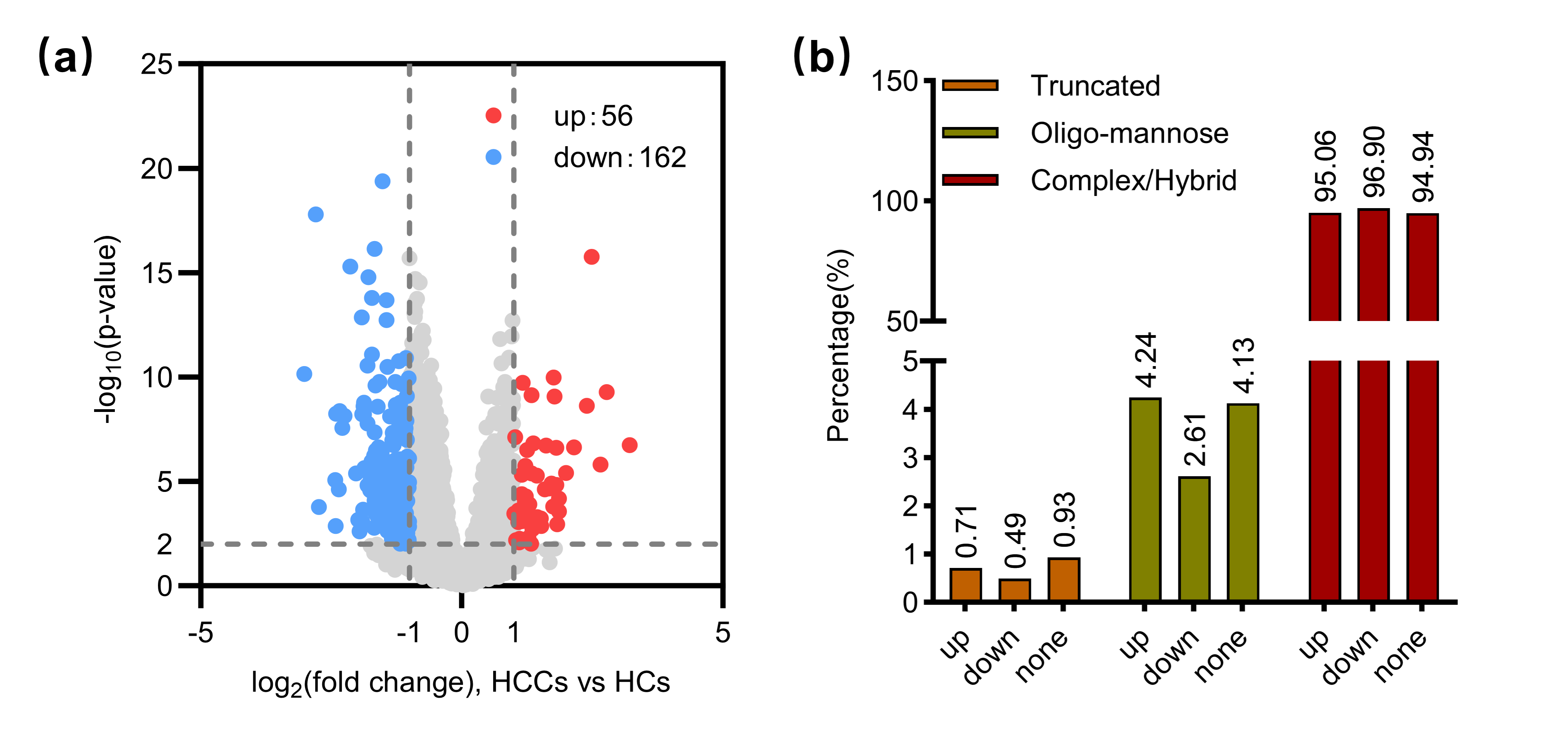


**Figure S5** (a) Volcano plot comparing site-specific glycans of HCCs versus HCs based on the discovery dataset (before-MBR). (b) Distribution of three types of glycans classified by glycan structures among up-regulated, down-regulated, and non-regulated site-specific glycans.


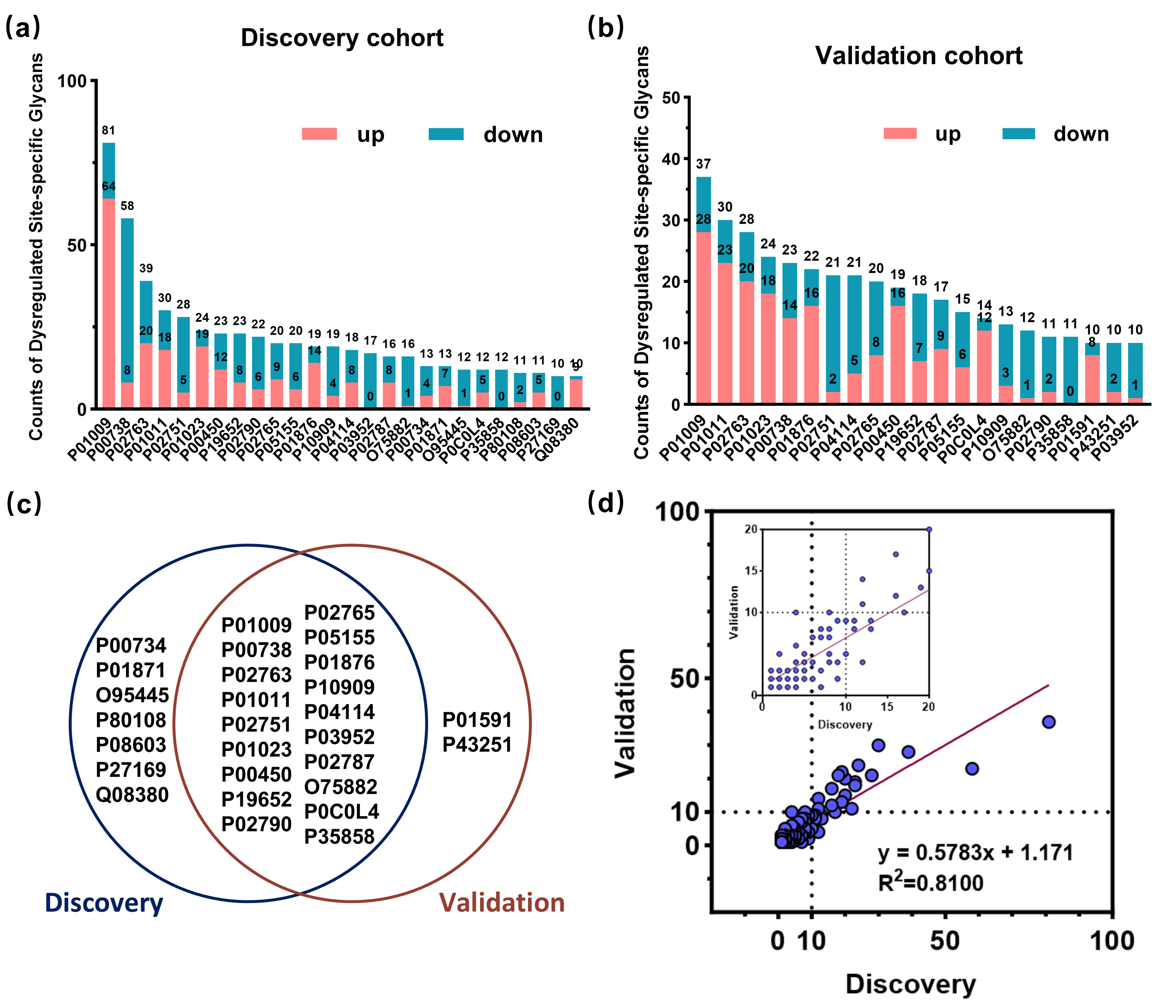


**Figure S6 Proteins prioritized by the protein-centric strategy in the discovery and validation cohorts.** (a) Distribution of up-regulated and down-regulated site-specific glycans among 26 proteins susceptible to aberrant glycosylation in the discovery cohort. (b) Distribution of up-regulated and down-regulated site-specific glycans among 21 proteins susceptible to aberrant glycosylation in the validation cohort. (c) Venn diagram displayed the distribution of proteins prioritized by the protein-centric strategy in the discovery and validation cohorts. (d) Correlation between the counts of dysregulated site-specific glycans among prioritized proteins both in the discovery and validation cohorts.


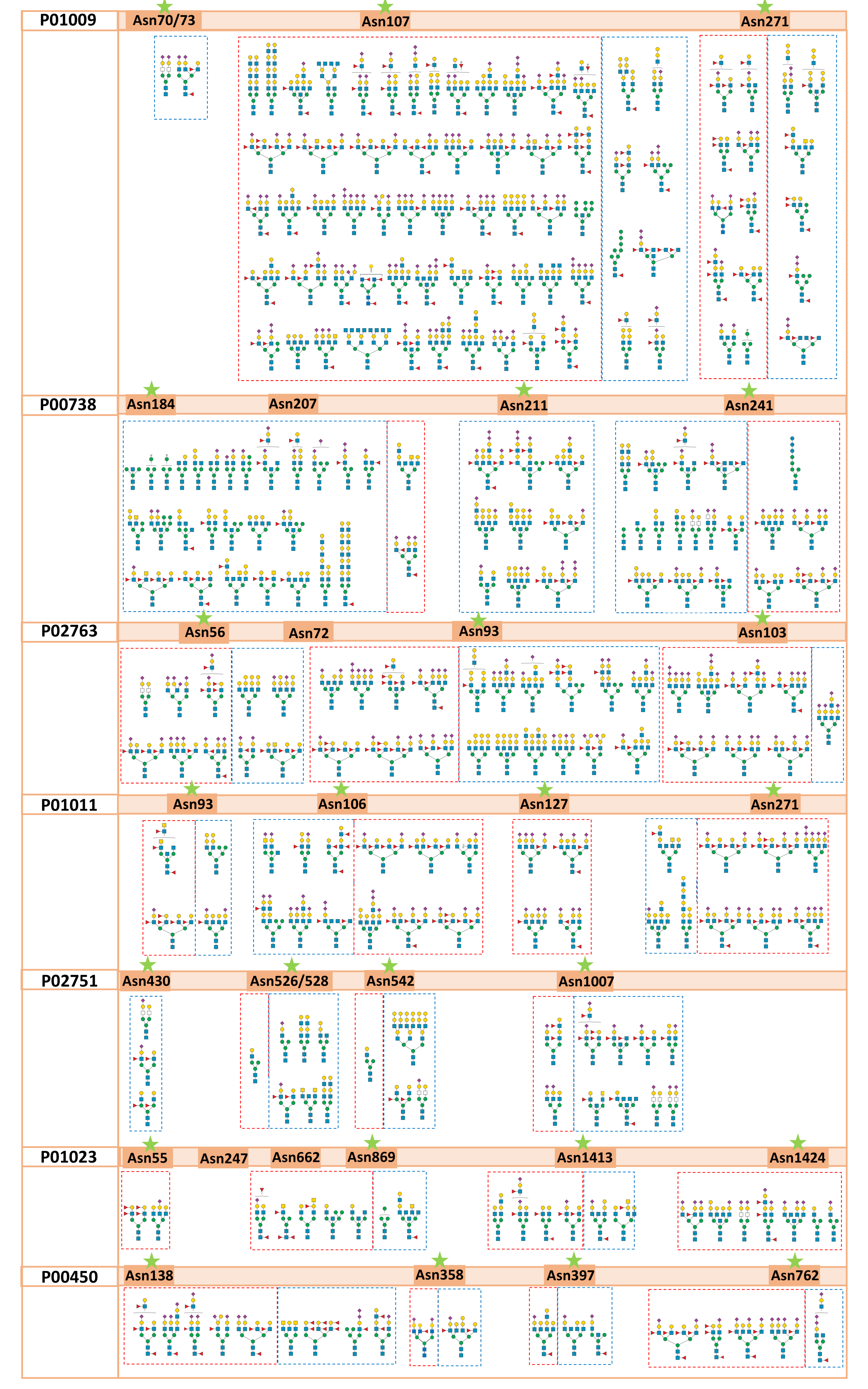


(Continued)


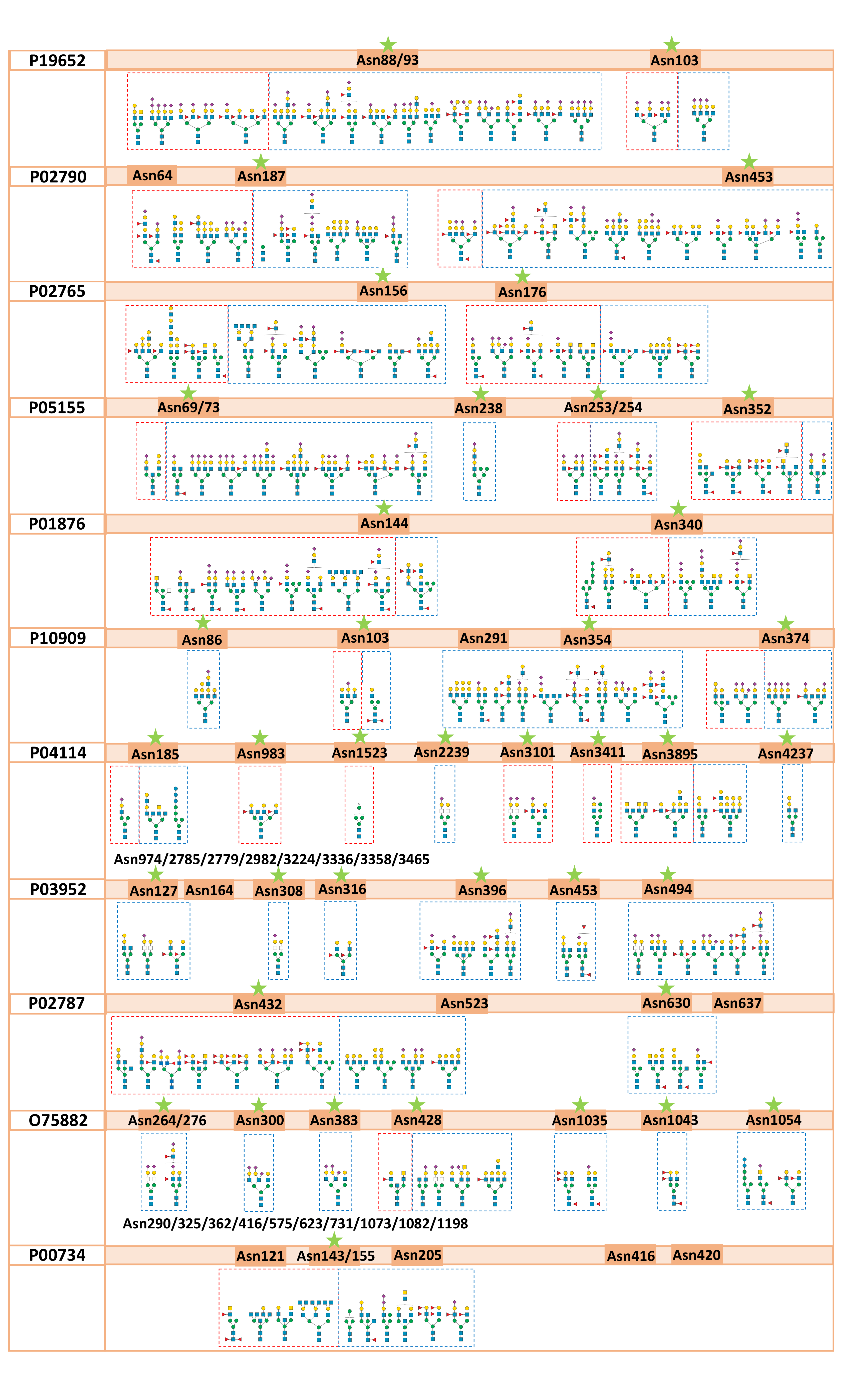


(Continued)


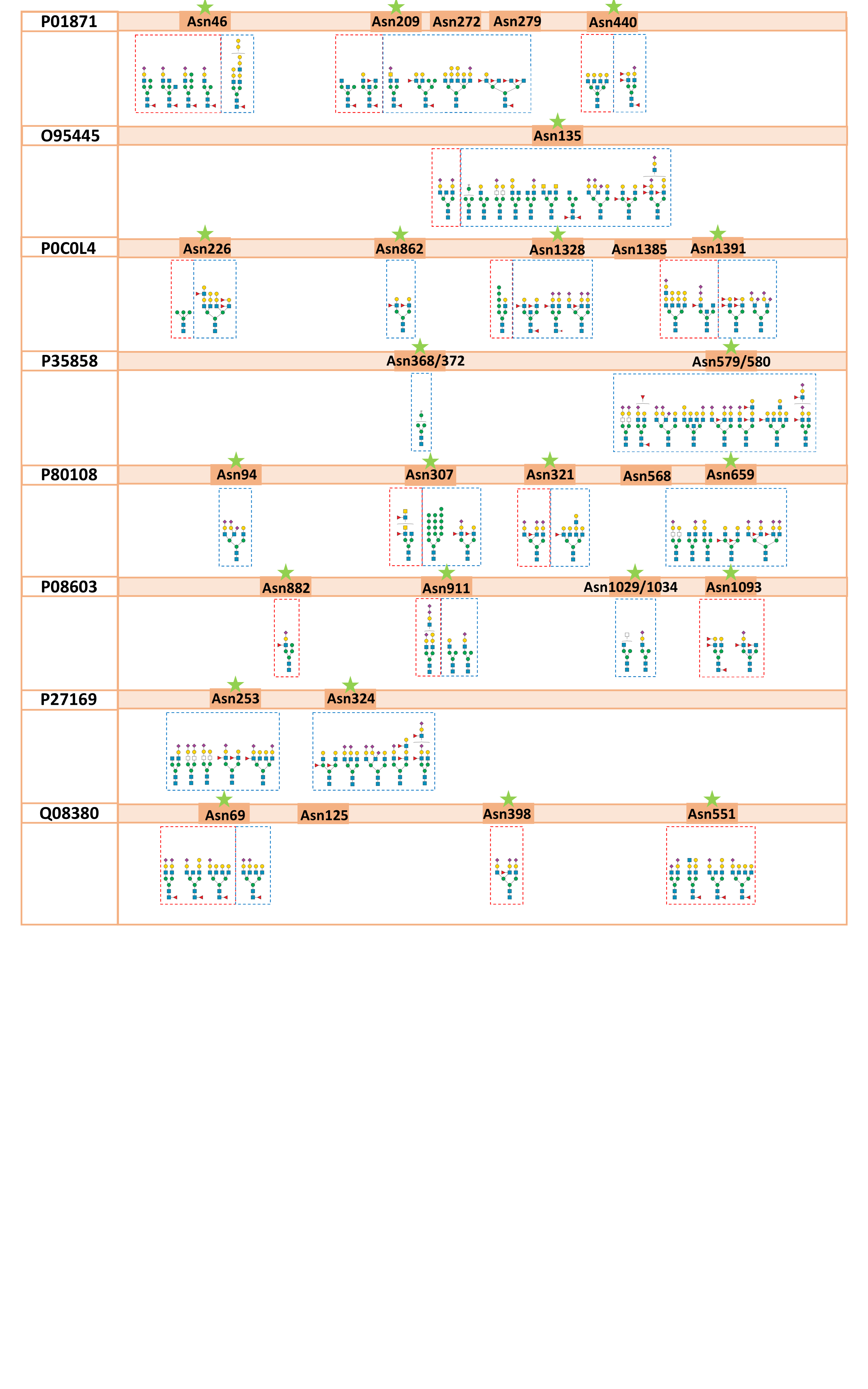


**Figure S7 The glycan structures corresponding to the dysregulated site-specific glycans of 26 proteins were screened from the discovery cohort.** The green asterisk: the glycosites containing dysregulated site-specific glycans. The red line: the up-regulated site-specific glycans. The blue lines: the down-regulated site-specific glycans. In addition, only one major glycanID structure corresponding to the site-specific glycan was presented in the figure. Some site-specific glycans were omitted as they were identified in the validation cohort. However, their quantification in the discovery cohort benefited from the MBR scheme.

The well studied type represented the protein has been purified from serum for focused analysis in a few studies. P00738 (Haptoglobin) is a well-studied glycoprotein, it was purified from serum in many studies^[4–8]^ to explore its potential as biomarker of HCC. Haptoglobin binds free hemoglobin to prevent oxidative damage, has antioxidant and anti-inflammatory effects, modulates immune responses, and serves as a key marker for hemolysis and inflammation. For Haptoglobin, 58 dysregulated site-specific glycans were found to distribute across Asn184, Asn211, and Asn241, corresponding to a total of 90 glycan structures (different glycan ID). We systematically presented the glycan structures corresponding to the dysregulated site-specific glycans of 26 proteins screened from the discovery cohort (Figure S7, Table S4). The moderate studied types could be divided into two parts, moderately studied^a)^ and moderately studied^b)^. Moderately studied^a)^ represented the protein has been purified for focused analysis in one study such as P01009(Alpha-1-antitrypsin),^[9]^ P02763 (Alpha-1-acid glycoprotein 1), and P19652(Alpha-1-acid glycoprotein 2).^[10]^ It should be noted that P01009 (Alpha-1-antitrypsin) exhibited the highest number of dysregulated site-specific glycans in both cohorts. This protein is primarily produced in the liver that plays a crucial role in protecting the lungs from enzyme-induced damage. Briefly, we quantified 81 dysregulated site-specific glycans from four glycosites in the discovery cohort, corresponding to a total of 129 aberrant glycosylation structures. And moderately studied^b)^ represented some experiments were conducted on the whole serum level, and the glycopeptide of this protein was finally selected as biomarker, such as P00450^[11]^ (Ceruloplasmin) and P27169^[12]^ (Serum paraoxonase/arylesterase 1). Finally, the minimally studied type, which meant glycopeptides of the protein only were listed as dysregulated glycopeptides in glycoproteomics studies without focused analysis. In addition, most of the proteins prioritized by this strategy were minimally studied in the intact glycopeptides level, including Fibronectin, Alpha-2-HS-glycoprotein, and Clusterin.


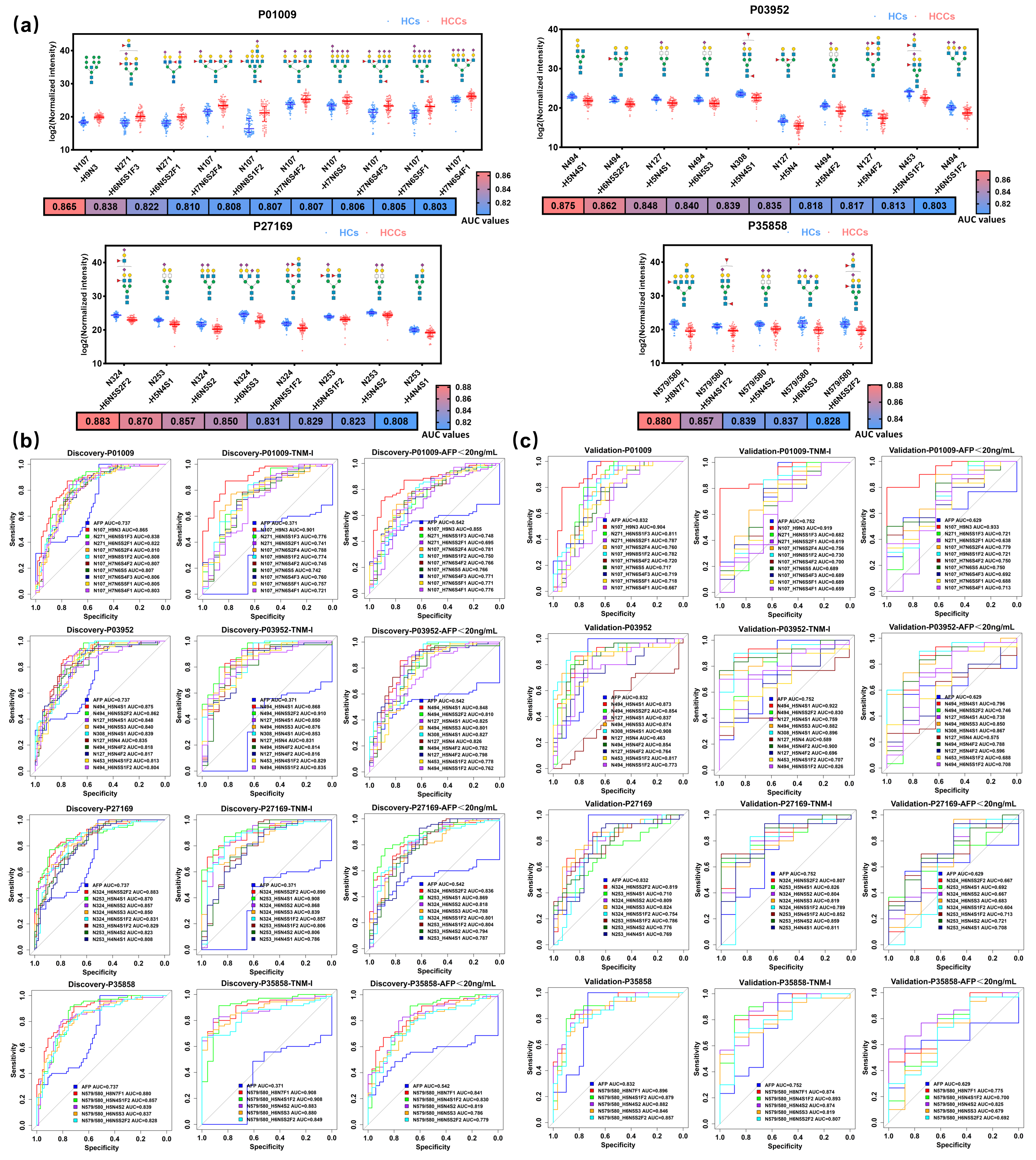


**Figure S8** **The glycan structures, abundance distribution, and diagnostic performance of site-specific glycans with the AUC values greater than 0.8 for P01009 (A1AT), P03952 (KLKB1), P27169 (PON1), and P35858 (ALS) proteins.** (a) The glycan structures, abundance distribution and the AUC values for HCC diagnosis of P01009, P03952, P27169 and P35858 proteins in the discovery cohort. (b) ROC curves of candidate biomarkers from four proteins and serum AFP for distinguishing: HCCs (n=70) versus HCs (n=70), TNM-I stage of HCCs (n=23) versus HCs (n=70) and AFP-negative HCCs (n=34) versus HCs (n=70) in the discovery cohort. (c) ROC curves of candidate biomarkers from four proteins and serum AFP for distinguishing: HCCs (n=30) versus HCs (n=30), TNM-I stage of HCCs (n=9) versus HCs (n=30) and AFP-negative of HCCs (n=8) versus HCs (n=30) in the validation cohort. For the P01009 protein, site-specific glycans with AUC > 0.8 were screened from both in the discovery and validation cohorts, excluding the N107_H8N7S2F4 due to missing values in the validation dataset, leaving 10 candidates for analysis.


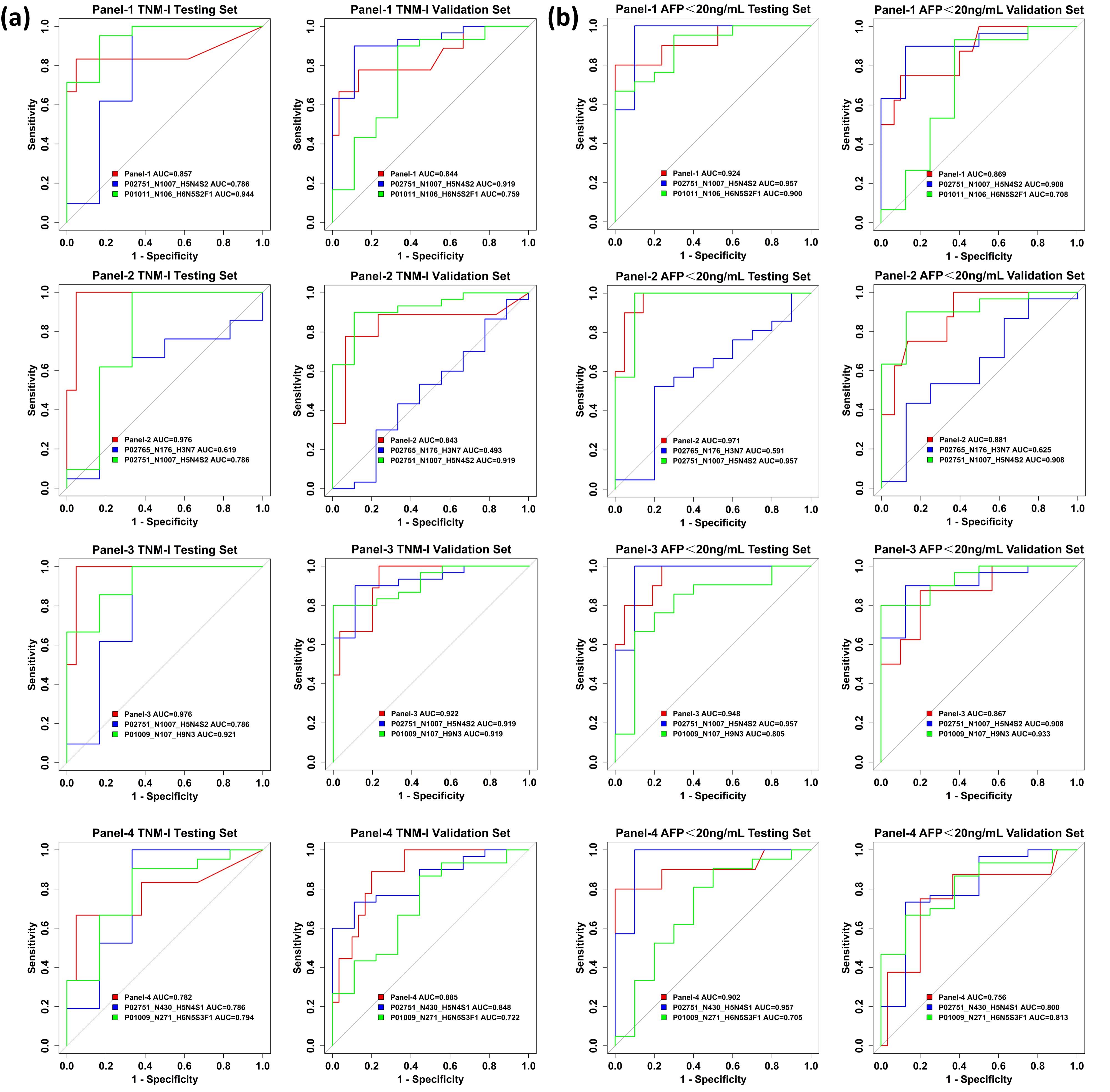


**Figure S9 ROC curves of four panels.** (a) Each panel comprised two site-specific glycans that were selected using a machine learning model for their complementary diagnostic performance in distinguishing TNM-I stage of HCCs (n=23) from the HCs (n=70), and validated in a separate cohort of TNM-I stage of HCCs (n=9) versus the HCs (n=30). (b) Each panel comprised two site-specific glycans that were selected using a machine learning model for their complementary diagnostic performance in distinguishing AFP-negative HCCs (n=34) from the HCs (n=70), and validated in a separate cohort of AFP-negative HCCs (n=8) versus the HCs (n=30).


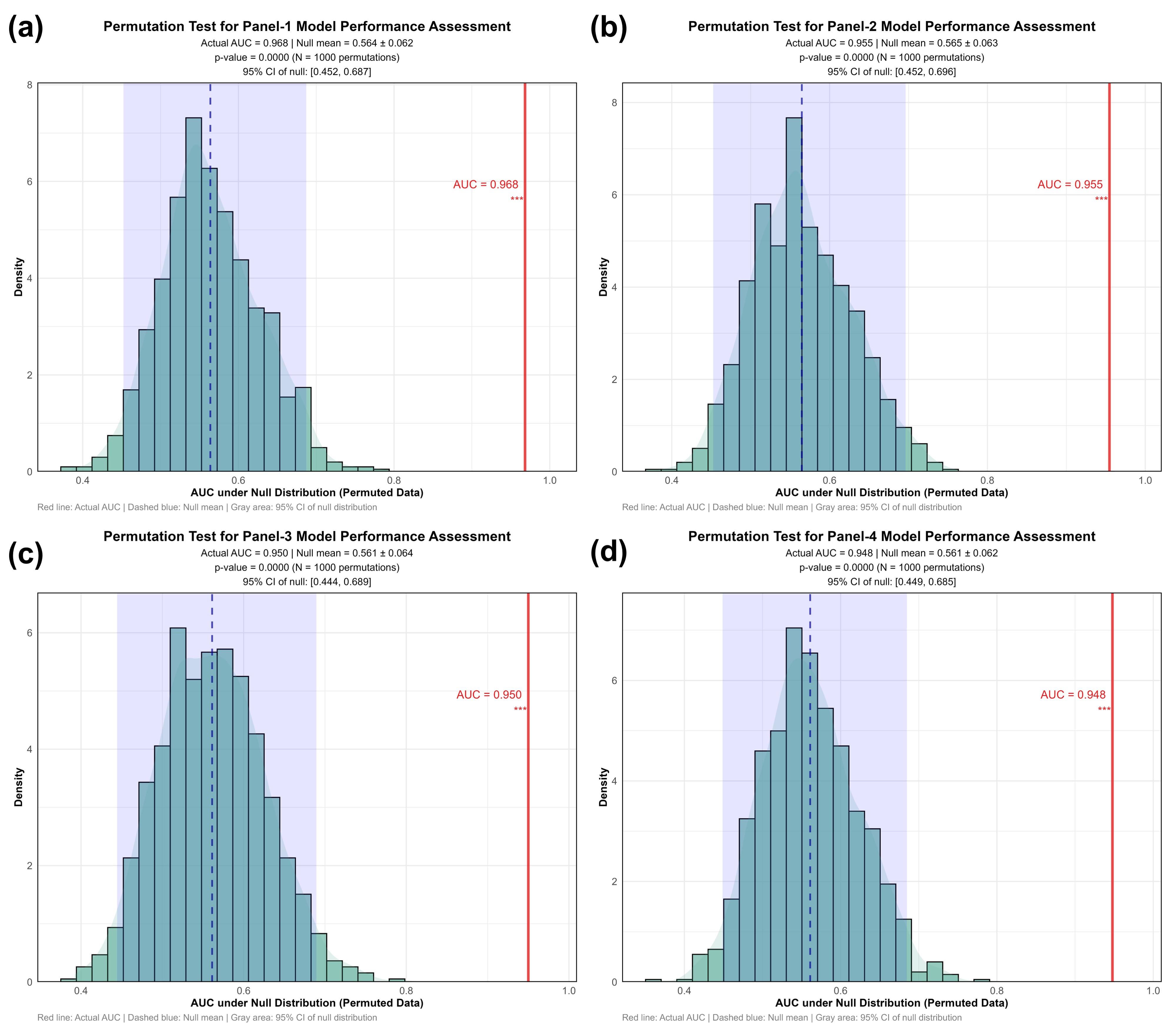


**Figure S10.** **Permutation test analysis comparing the performance of four random forest panels in discriminating HCCs versus HCs.** The null distribution from 1000 permutations is shown as a green histogram. Red vertical line indicates the true AUC value; the blue dashed line indicates the mean of the null distribution. Shaded area represents the 95% confidence interval of the null distribution. The clear separation (p < 0.001) indicates statistically significant performance.

To further evaluate model stability and significance, we demonstrated the robustness of the final four selected panels by presenting their cross-validation, and the evaluation of model significance results by presenting the permutation test analysis (Figure S10, Table S9). We performed a 5-fold cross-validation (mtry=1, 500 trees) for the constructed random forest model in the train-set data using the R caret package, reporting the mean AUC values, indicating excellent and stable discriminative ability. Additionally, we conducted 1000 permutation tests to establish a null distribution in the testing-set data, calculated the statistical significance (p-value) of the model performance, and generated an intuitive permutation test plot to evaluate model significance. The permutation test demonstrated strong statistical significance for our model (p < 0.001). The clear separation between the actual AUC (red vertical line) and the null distribution (green histogram) indicates that the observed performance is highly unlikely to occur by chance, confirming that the model has learned biologically meaningful patterns rather than fitting to random noise.

Table S1. Clinical characteristics of the discovery and validation cohorts.

| **Characteristic** | **Discovery cohort** | | | **Validation cohort** | |
| --- | --- | --- | --- | --- | --- |
|  | **HCCs (n=70)** | **HCs (n=70)** | **HCCs (n=30)** | | **HCs (n=30)** |
| Gender  (male/female) | 56/14 | 56/14 | 23/7 | | 23/7 |
| Age  (mean ± SD) | 60.9±9.4 | 60.2±10.2 | 62.4 ±10.4 | | 62.6 ±10.7 |
| Serum AFP, ng mL^-1^ (mean ± SD) | 3698.4±17719.3 | 7.7±6.9 | 1769.1±3891.6 | | 8.5±6.5 |
| TNM  TNM I/II/III/IV | 23/20/20/7 | NA | 9/9/9/3 | | NA |

Table S2. The proteins corresponding to 1,038 dysregulated site-specific glycans (SSG) are sorted in descending order based on the number of dysregulated site-specific glycans they contained, and the proteins containing at least 9 dysregulated site-specific glycans were displayed in the list.

| # | Protein ID | counts of Dysregulated SSG |
| --- | --- | --- |
| 1 | P01009 | 81 |
| 2 | P00738 | 58 |
| 3 | P02763 | 39 |
| 4 | P01011 | 30 |
| 5 | P02751 | 28 |
| 6 | P01023 | 24 |
| 7 | P00450 | 23 |
| 8 | P19652 | 23 |
| 9 | P02790 | 22 |
| 10 | P02765 | 20 |
| 11 | P05155 | 20 |
| 12 | P01876 | 19 |
| 13 | P10909 | 19 |
| 14 | P04114 | 18 |
| 15 | P03952 | 17 |
| 16 | P02787 | 16 |
| 17 | O75882 | 16 |
| 18 | P00734 | 13 |
| 19 | P01871 | 13 |
| 20 | O95445 | 12 |
| 21 | P0C0L4 | 12 |
| 22 | P35858 | 12 |
| 23 | P80108 | 11 |
| 24 | P08603 | 11 |
| 25 | P27169 | 10 |
| 26 | Q08380 | 10 |
| 27 | Q14624 | 9 |
| 28 | Q96PD5 | 9 |
| 29 | P05090 | 9 |
| … | … | … |

Table S3. 26 proteins prioritized by the protein-centric strategy in the discovery cohort.

| Uniport ID | Protein | Protein abbreviation | Gene |
| --- | --- | --- | --- |
| P01009 | Alpha-1-antitrypsin | A1AT | SERPINA1 |
| P00738 | Haptoglobin | HPT | HP |
| P02763 | Alpha-1-acid glycoprotein 1 | A1AG1 | ORM1 |
| P01011 | Alpha-1-antichymotrypsin | AACT | SERPINA3 |
| P02751 | Fibronectin | FN1 | FINC |
| P01023 | Alpha-2-macroglobulin | A2MG | A2M |
| P00450 | Ceruloplasmin | CERU | CP |
| P19652 | Alpha-1-acid glycoprotein 2 | A1AG2 | ORM2 |
| P02790 | Hemopexin | HEMO | HPX |
| P02765 | Alpha-2-HS-glycoprotein | FETUA | AHSG |
| P05155 | Plasma protease C1 inhibitor | IC1 | SERPING1 |
| P01876 | Immunoglobulin heavy constant alpha 1 | IGHA1 | IGHA1 |
| P10909 | Clusterin | CLUS | CLU |
| P04114 | Apolipoprotein B-100 | APOB | APOB |
| P03952 | Plasma kallikrein | KLKB1 | KLKB1 |
| P02787 | Serotransferrin | TRFE | TF |
| O75882 | Attractin | ATRN | ATRN |
| P00734 | Prothrombin | THRB | F2 |
| P01871 | Immunoglobulin heavy constant mu | IGHM | IGHM |
| O95445 | Apolipoprotein M | APOM | APOM |
| P0C0L4 | Complement C4-A | CO4A | C4A |
| P35858 | Insulin-like growth factor-binding protein complex acid labile subunit | ALS | IGFALS |
| P80108 | Phosphatidylinositol-glycan-specific phospholipase D | PHLD | GPLD1 |
| P08603 | Complement factor H | CFAH | CFH |
| P27169 | Serum paraoxonase/arylesterase 1 | PON1 | PON1 |
| Q08380 | Galectin-3-binding protein | LG3BP | LGALS3BP |

Table S4. 21 proteins prioritized by the protein-centric strategy in the validation cohort.

| Uniport ID | Protein | Protein abbreviation | Gene |
| --- | --- | --- | --- |
| P01009 | Alpha-1-antitrypsin | A1AT | SERPINA1 |
| P01011 | Alpha-1-antichymotrypsin | AACT | SERPINA3 |
| P02763 | Alpha-1-acid glycoprotein 1 | A1AG1 | ORM1 |
| P01023 | Alpha-2-macroglobulin | A2MG | A2M |
| P00738 | Haptoglobin | HPT | HP |
| P01876 | Immunoglobulin heavy constant alpha 1 | IGHA1 | IGHA1 |
| P02751 | Fibronectin | FN1 | FINC |
| P04114 | Apolipoprotein B-100 | APOB | APOB |
| P02765 | Alpha-2-HS-glycoprotein | FETUA | AHSG |
| P00450 | Ceruloplasmin | CERU | CP |
| P19652 | Alpha-1-acid glycoprotein 2 | A1AG2 | ORM2 |
| P02787 | Serotransferrin | TRFE | TF |
| P05155 | Plasma protease C1 inhibitor | IC1 | SERPING1 |
| P0C0L4 | Complement C4-A | CO4A | C4A |
| P10909 | Clusterin | CLUS | CLU |
| O75882 | Attractin | ATRN | ATRN |
| P02790 | Hemopexin | HEMO | HPX |
| P35858 | Insulin-like growth factor-binding protein complex acid labile subunit | ALS | IGFALS |
| P01591 | Immunoglobulin J chain | IGJ | JCHAIN |
| P43251 | Biotinidase | BTD | BTD |
| P03952 | Plasma kallikrein | KLKB1 | KLKB1 |

Table S5. The N-glycosites containing dysregulated site-specific glycans were identified among the 26 proteins prioritized by the protein-centric strategy in the discovery cohort.

| Uniport ID | Protein | N-glycosites containing dysregulated site-specific glycans |
| --- | --- | --- |
| P01009 | Alpha-1-antitrypsin | Asn70/73, Asn107, Asn271 |
| P00738 | Haptoglobin | Asn184, Asn211, Asn241 |
| P02763 | Alpha-1-acid glycoprotein 1 | Asn56, Asn93, Asn103 |
| P01011 | Alpha-1-antichymotrypsin | Asn93, Asn106, Asn127, Asn271 |
| P02751 | Fibronectin | Asn430, Asn526/528, Asn542, Asn1007 |
| P01023 | Alpha-2-macroglobulin | Asn55, Asn869, Asn1413, Asn1424 |
| P00450 | Ceruloplasmin | Asn138, Asn358, Asn397, Asn762 |
| P19652 | Alpha-1-acid glycoprotein 2 | Asn88/93, Asn103 |
| P02790 | Hemopexin | Asn187, Asn453 |
| P02765 | Alpha-2-HS-glycoprotein | Asn156, Asn176 |
| P05155 | Plasma protease C1 inhibitor | Asn69/73, Asn238, Asn253/254, Asn352 |
| P01876 | Immunoglobulin heavy constant alpha 1 | Asn144, Asn340 |
| P10909 | Clusterin | Asn86, Asn103, Asn354, Asn374 |
| P04114 | Apolipoprotein B-100 | Asn185, Asn983, Asn1523, Asn2239, Asn3101, Asn3411, Asn3895, Asn4237 |
| P03952 | Plasma kallikrein | Asn127, Asn308, Asn316, Asn396, Asn453, Asn494 |
| P02787 | Serotransferrin | Asn432, Asn630 |
| O75882 | Attractin | Asn264/276, Asn300, Asn383, Asn428, Asn1035, Asn1043, Asn1054 |
| P00734 | Prothrombin | Asn143/155 |
| P01871 | Immunoglobulin heavy constant mu | Asn46, Asn209, Asn440 |
| O95445 | Apolipoprotein M | Asn135 |
| P0C0L4 | Complement C4-A | Asn226, Asn862, Asn1328, Asn1391 |
| P35858 | Insulin-like growth factor-binding protein complex acid labile subunit | Asn368/372, Asn579/580 |
| P80108 | Phosphatidylinositol-glycan-specific phospholipase D | Asn94, Asn307, Asn321, Asn659 |
| P08603 | Complement factor H | Asn882, Asn911, Asn1029/1034, Asn1093 |
| P27169 | Serum paraoxonase/arylesterase 1 | Asn253, Asn324 |
| Q08380 | Galectin-3-binding protein | Asn69, Asn398, Asn551 |

Table S6. The diagnostic performance of dysregulated site-specific glycans among those five proteins in the discovery cohort.

| Proteins | Site-specific glycans | Expression | AUC |
| --- | --- | --- | --- |
| P01009 | Hex(9)HexNAc(3) P01009@107 | up | 0.865 |
|  | Hex(6)HexNAc(5)NeuAc(1)Fuc(3) P01009@271 | up | 0.838 |
|  | Hex(6)HexNAc(5)NeuAc(2)Fuc(1) P01009@271 | up | 0.822 |
|  | Hex(7)HexNAc(6)NeuAc(2)Fuc(4) P01009@107 | up | 0.810 |
|  | Hex(9)HexNAc(8)NeuAc(1)Fuc(2) P01009@107 | up | 0.808 |
|  | Hex(7)HexNAc(6)NeuAc(4)Fuc(2) P01009@107 | up | 0.807 |
|  | Hex(7)HexNAc(6)NeuAc(5) P01009@107 | up | 0.807 |
|  | Hex(7)HexNAc(6)NeuAc(4)Fuc(3) P01009@107 | up | 0.806 |
|  | Hex(7)HexNAc(6)NeuAc(5)Fuc(1) P01009@107 | up | 0.805 |
|  | Hex(8)HexNAc(7)NeuAc(2)Fuc(4) P01009@107 | up | 0.804 |
|  | Hex(7)HexNAc(6)NeuAc(4)Fuc(1) P01009@107 | up | 0.803 |
| P02751^a)^ | Hex(5)HexNAc(4)NeuAc(2) P02751@1007 | down | 0.917 |
|  | Hex(5)HexNAc(4)Fuc(2) P02751@542 | down | 0.908 |
|  | Hex(5)HexNAc(4)NeuAc(1) P02751@430 | down | 0.900 |
|  | Hex(5)HexNAc(4)NeuAc(1)Fuc(2) P02751@526 | down | 0.891 |
|  | Hex(6)HexNAc(4)NeuAc(1)Fuc(1) P02751@1007 | down | 0.872 |
|  | Hex(5)HexNAc(4)NeuAc(1)Fuc(2) P02751@528 | down | 0.871 |
|  | Hex(5)HexNAc(4)NeuAc(1) P02751@1007 | down | 0.857 |
|  | Hex(5)HexNAc(4)NeuAc(1) P02751@542 | down | 0.850 |
|  | Hex(5)HexNAc(4)Fuc(2) P02751@430 | down | 0.849 |
|  | Hex(3)HexNAc(7) P02751@528 | down | 0.805 |
| P03952 | Hex(5)HexNAc(4)NeuAc(1) P03952@494 | down | 0.875 |
|  | Hex(6)HexNAc(5)NeuAc(2)Fuc(2) P03952@494 | down | 0.862 |
|  | Hex(5)HexNAc(4)NeuAc(1) P03952@127 | down | 0.848 |
|  | Hex(6)HexNAc(5)NeuAc(3) P03952@494 | down | 0.840 |
|  | Hex(5)HexNAc(4)NeuAc(1) P03952@308 | down | 0.839 |
|  | Hex(5)HexNAc(4) P03952@127 | down | 0.835 |
|  | Hex(5)HexNAc(4)Fuc(2) P03952@494 | down | 0.818 |
|  | Hex(5)HexNAc(4)Fuc(2) P03952@127 | down | 0.817 |
|  | Hex(5)HexNAc(4)NeuAc(1)Fuc(2) P03952@453 | down | 0.813 |
|  | Hex(6)HexNAc(5)NeuAc(1)Fuc(2) P03952@494 | down | 0.803 |
| P27169 | Hex(6)HexNAc(5)NeuAc(2)Fuc(2) P27169@324 | down | 0.883 |
|  | Hex(5)HexNAc(4)NeuAc(1) P27169@253 | down | 0.870 |
|  | Hex(6)HexNAc(5)NeuAc(2) P27169@324 | down | 0.857 |
|  | Hex(6)HexNAc(5)NeuAc(3) P27169@324 | down | 0.850 |
|  | Hex(6)HexNAc(5)NeuAc(1)Fuc(2) P27169@324 | down | 0.831 |
|  | Hex(5)HexNAc(4)NeuAc(1)Fuc(2) P27169@253 | down | 0.829 |
|  | Hex(5)HexNAc(4)NeuAc(2) P27169@253 | down | 0.823 |
|  | Hex(4)HexNAc(4)NeuAc(1) P27169@253 | down | 0.808 |
| P35858^b)^ | Hex(8)HexNAc(7)Fuc(1) P35858@579;P35858@580 | down | 0.880 |
|  | Hex(5)HexNAc(4)NeuAc(1)Fuc(2) P35858@579;P35858@580 | down | 0.857 |
|  | Hex(5)HexNAc(4)NeuAc(2) P35858@579;P35858@580 | down | 0.839 |
|  | Hex(6)HexNAc(5)NeuAc(3) P35858@579;P35858@580 | down | 0.837 |
|  | Hex(6)HexNAc(5)NeuAc(2)Fuc(2) P35858@579;P35858@580 | down | 0.828 |

Two site-specific glycans in protein^a)^ and protein^b)^ were excluded as duplicate features because of their localization to the same protein and coverage of overlapping glycosylation sites, confirming they represented the same feature.

Table S7. The four combinations with the AUC values greater than 0.940 in both the testing and validation sets based on machine learning models.

| Panel | Feature-1 | Feature-2 | AUC  Testing- sets | AUC  Validation-sets |
| --- | --- | --- | --- | --- |
| Panel-1 | Hex(5)HexNAc(4)NeuAc(2)  P02751@1007 | Hex(6)HexNAc(5)NeuAc(2)Fuc(1)  P01011@106 | 0.968 | 0.952 |
| Panel-2 | Hex(3)HexNAc(7)  P02765@176 | Hex(5)HexNAc(4)NeuAc(2)  P02751@1007 | 0.955 | 0.952 |
| Panel-3 | Hex(5)HexNAc(4)NeuAc(2)  P02751@1007 | Hex(9)HexNAc(3)  P01009@107 | 0.950 | 0.973 |
| Panel-4 | Hex(5)HexNAc(4)NeuAc(1)  P02751@430 | Hex(6)HexNAc(5)NeuAc(3)Fuc(1)  P01009@271 | 0.948 | 0.951 |

Table S9. Performance validation of the four biomarker panels. The mean AUC represents the average performance from the 5-fold cross-validation, and asterisks denote statistical significance levels from permutation tests (***p < 0.001).

| **Panel** | **Feature-1** | **Feature-2** | **5-CV_results$ROC**  **(mean±sd)** | **Significance** |
| --- | --- | --- | --- | --- |
| Panel-1 | P02751_N1007_H5N4S2 | P01011_N106_H6N5S2F1 | 0.9492 ± 0.0574 | *** p < 0.001 |
| Panel-2 | P02765_N176_H3N7 | P02751_N1007_ H5N4S2 | 0.9027 ± 0.0828 | *** p < 0.001 |
| Panel-3 | P02751_N1007_ H5N4S2 | P01009_N107_H9N3 | 0.9726 ± 0.0213 | *** p < 0.001 |
| Panel-4 | P02751_N430_ H5N4S1 | P01009_N271_H6N5S3F1 | 0.9332 ± 0.0543 | *** p < 0.001 |

**References**

[1] Zhu J, Warner E, Parikh ND, Lubman DM. Glycoproteomic markers of hepatocellular carcinoma-mass spectrometry based approaches. *Mass Spectrometry Reviews*. 2019;38(3):265-290. doi:[10.1002/mas.21583](https://doi.org/10.1002/mas.21583)

[2] Blomme B, Van Steenkiste C, Callewaert N, Van Vlierberghe H. Alteration of protein glycosylation in liver diseases. *Journal of Hepatology*. 2009;50(3):592-603. doi:[10.1016/j.jhep.2008.12.010](https://doi.org/10.1016/j.jhep.2008.12.010)

[3] Butaye E, Somers N, Grossar L, et al. Systematic review: Glycomics as diagnostic markers for hepatocellular carcinoma. *Alimentary Pharmacology & Therapeutics*. 2024;59(1):23-38. doi:[10.1111/apt.17748](https://doi.org/10.1111/apt.17748)

[4] Lin Y, Zhu J, Zhang J, et al. Glycopeptides with sialyl lewis antigen in serum haptoglobin as candidate biomarkers for nonalcoholic steatohepatitis hepatocellular carcinoma using a higher-energy collision-induced dissociation parallel reaction monitoring-mass spectrometry method. *ACS Omega*. 2022;7(26):22850-22860. doi:[10.1021/acsomega.2c02600](https://doi.org/10.1021/acsomega.2c02600)

[5] Gutierrez Reyes CD, Huang Y, Atashi M, et al. PRM-MS Quantitative Analysis of Isomeric N-Glycopeptides Derived from Human Serum Haptoglobin of Patients with Cirrhosis and Hepatocellular Carcinoma. *Metabolites*. 2021;11(8):563. doi:[10.3390/metabo11080563](https://doi.org/10.3390/metabo11080563)

[6] Pradita T, Chen YJ, Su TH, Chang KH, Chen PJ, Chen YJ. Data independent acquisition mass spectrometry enhanced personalized glycosylation profiling of haptoglobin in hepatocellular carcinoma. *J Proteome Res*. 2024;23(8):3571-3584. doi:[10.1021/acs.jproteome.4c00227](https://doi.org/10.1021/acs.jproteome.4c00227)

[7] Zhu J, Chen Z, Zhang J, et al. Differential quantitative determination of site-specific intact N-glycopeptides in serum haptoglobin between hepatocellular carcinoma and cirrhosis using LC-EThcD-MS/MS. *J Proteome Res*. 2019;18(1):359-371. doi:[10.1021/acs.jproteome.8b00654](https://doi.org/10.1021/acs.jproteome.8b00654)

[8] Kohansal-Nodehi M, Swiatek-de Lange M, Kroeniger K, et al. Discovery of a haptoglobin glycopeptides biomarker panel for early diagnosis of hepatocellular carcinoma. *Front Oncol*. 2023;13. doi:[10.3389/fonc.2023.1213898](https://doi.org/10.3389/fonc.2023.1213898)

[9] Yin H, Zhu J, Wang M, Yao ZP, Lubman DM. Quantitative analysis of α-1-antitrypsin glycosylation isoforms in HCC patients using LC-HCD-PRM-MS. *Anal Chem*. 2020;92(12):8201-8208. doi:[10.1021/acs.analchem.0c00420](https://doi.org/10.1021/acs.analchem.0c00420)

[10] Zhang D, Huang J, Luo D, Feng X, Liu Y, Liu Y. Glycosylation change of alpha-1-acid glycoprotein as a serum biomarker for hepatocellular carcinoma and cirrhosis. *Biomarkers in Medicine*. 2017;11(5):423-430. doi:[10.2217/bmm-2016-0284](https://doi.org/10.2217/bmm-2016-0284)

[11] Lin Y, Zhang J, Arroyo A, Singal AG, Parikh ND, Lubman DM. A fucosylated glycopeptide as a candidate biomarker for early diagnosis of NASH hepatocellular carcinoma using a stepped HCD method and PRM evaluation. *Front Oncol*. 2022;12. doi:[10.3389/fonc.2022.818001](https://doi.org/10.3389/fonc.2022.818001)

[12] Cao X, Cao Z, Shao Y, et al. Analysis of serum paraoxonase 1 using mass spectrometry and lectin immunoassay in patients with alpha-fetoprotein negative hepatocellular carcinoma. *Front Oncol*. 2021;11. doi:[10.3389/fonc.2021.651421](https://doi.org/10.3389/fonc.2021.651421)
